# Drivers of the composition of active rhizosphere bacterial communities in temperate grasslands

**DOI:** 10.1038/s41396-019-0543-4

**Published:** 2019-10-28

**Authors:** Selma Vieira, Johannes Sikorski, Sophie Dietz, Katharina Herz, Marion Schrumpf, Helge Bruelheide, Dierk Scheel, Michael W. Friedrich, Jörg Overmann

**Affiliations:** 1 Leibniz Institute DSMZ - German Collection of Microorganisms and Cell Cultures, Inhoffenstraße 7B, 38124 Braunschweig, Germany; 2 Leibniz Institute of Plant Biochemistry, Weinberg 3, 06120 Halle (Saale), Germany; 3 Institute of Biology/Geobotany and Botanical Garden, Martin Luther University Halle-Wittenberg, Am Kirchtor 1, 06108 Halle (Saale), Germany; 4 Max Planck Institute for Biogeochemistry, Hans-Knöll-Straße 10, 07745 Jena, Germany; 5 German Centre for Integrative Biodiversity Research (iDiv) Halle-Jena-Leipzig, Deutscher Platz 5e, 04103 Leipzig, Germany; 6 Faculty of Biology/Chemistry and Center for Marine Environmental Science (MARUM), University of Bremen, Leobener Straße 3, 28359 Bremen, Germany; 7 Braunschweig University of Technology, Universitätsplatz 2, 38106 Braunschweig, Germany

**Keywords:** Microbial ecology, Biodiversity

## Abstract

The active bacterial rhizobiomes and root exudate profiles of phytometers of six plant species growing in central European temperate grassland communities were investigated in three regions located up to 700 km apart, across diverse edaphic conditions and along a strong land use gradient. The recruitment process from bulk soil communities was identified as the major direct driver of the composition of active rhizosphere bacterial communities. Unexpectedly, the effect of soil properties, particularly soil texture, water content, and soil type, strongly dominated over plant properties and the composition of polar root exudates of the primary metabolism. While plant species-specific selection of bacteria was minor, the RNA-based composition of active rhizosphere bacteria substantially differed between rhizosphere and bulk soil. Although other variables could additionally be responsible for the consistent enrichment of particular bacteria in the rhizosphere, distinct bacterial OTUs were linked to the presence of specific polar root exudates independent of individual plant species. Our study also identified numerous previously unknown taxa that are correlated with rhizosphere dynamics and hence represent suitable targets for future manipulations of the plant rhizobiome.

## Introduction

The rhizosphere comprises the mm-thin soil layer surrounding plant roots and is characterized by high concentrations of plant-derived organic exudates, the release of root cap mucilage and of root border cells. Environmental conditions for rhizosphere bacteria differ from those in bulk soil with respect to pH, water, oxygen, and nutrient content, the composition and concentrations of bacterial growth substrates, and the presence of antimicrobial compounds and plant hormones [1, 2]. Compared with bulk soil, rhizosphere bacterial communities show elevated densities, larger cell sizes [2–4], increased microbial activity [5, 6], and higher turnover rates. As a result, their cellular production contributes significantly to the global carbon cycle [5]. Rhizosphere bacterial communities also affect plant growth, induce systemic resistance or directly inhibit plant pathogens [7–10], and impact nutrient cycling [11] through the solubilization of soil minerals [12]. Conditions within the rhizosphere select for specific microbial populations [13–17], which often have a decreased species richness and evenness [16–19] although contradictory findings have been reported [14, 20, 21].

Root exudates can differ between plant species, their different growth stages, and even between cultivars of the same plant species [22–28], and select for rhizosphere bacteria with matching substrate uptake preferences [29, 30]. As a result, the rhizobiome composition can differ between plant species [31–33] or between plant genotypes [8, 18, 20, 22]. To date, the majority of rhizobiome studies focused on model or crop plants grown in microcosms or monospecific culture [17, 18, 23, 32, 34–37]. Non-crop plant species, particularly in complex natural communities, have been studied only rarely [15, 26, 28, 38]. In addition to plant species, edaphic factors or other environmental conditions may have dominant effects on the composition of rhizosphere bacterial communities [18–20, 35, 37, 39]. While some studies covered the effects of both, plant species and soil types [40, 41], comparative studies of the different potential drivers of the composition of the active rhizobiome are rare [42].

The aim of the current study was to determine the influence of plant species, soil conditions, and polar root exudate composition on the composition of active rhizosphere bacterial communities in natural temperate grasslands under different edaphic and climatic conditions, and along a land use gradient. Root-associated bacteria are typically more active than those in the surrounding bulk soil. In most bacteria, changes in the cellular ribosome content are proportional to the growth rate [43–45]. Within bacterial communities, active taxa can therefore be assessed by high throughput sequencing of cDNA generated from the extracted 16S rRNA transcripts. This active fraction is controlled by different environmental variables than total community composition (as determined by 16S rRNA gene sequencing) [46, 47]. Compositional changes in rRNA transcripts occur in response to carbon and nutrient additions in soils [48] and correlate with changes in important ecosystem functions such as CO_2_ production, unlike total community composition [49]. Furthermore, highly active and biogeochemically relevant microbial taxa may be rare or even absent from DNA-based sequence inventories of microbial communities [46, 50, 51]. Finally, community analyses based on rRNA have yielded a significantly higher β-diversity for rhizosphere samples than DNA-based analyses [21], allowing a better differentiation of the effects of environmental drivers. In the present study, we analyzed active bacterial communities in the rhizosphere of phytometer plants of three forb and three grass species in a multifactorial approach.

## Material and methods

### Study area and sampling

The present study was performed in the framework of the Biodiversity Exploratories (www.biodiversity-exploratories.de). These large-scale, long-term ecological research sites were previously established in the biosphere reserve Schorfheide-Chorin (Brandenburg, north-eastern Germany), the national park Hainich and its surroundings in Thuringia (central Germany), and the biosphere reserve Schwäbische Alb in Baden-Württemberg (south-western Germany) [52].

Because they are perennial and based on their high natural abundance, *Plantago lanceolata* (Plantaginaceae), *Achillea millefolium* (Asteraceae), and *Ranunculus acris* (Ranunculaceae) were selected as forb species and *Dactylis glomerata*, *Arrhenatherum elatius,* and *Alopecurus pratensis* (all from the Poaceae family) as grass species. In order to assess the effects of plant species and environmental conditions under highly controlled and reproducible conditions, we employed plant phytometers. A phytometer is a plant that has been grown under controlled conditions and is then exposed to specific environments [53]. Raising and transplanting uniform plants ensures that all plant specimens in all plots have the same age and progeny, minimizing confounding factors. Plant phytometers were initially grown axenically from seeds collected from and around the sites, in a sterile sand/humus mixture in a greenhouse and were transplanted to the study sites in May to early June 2014 (for raising conditions, planting process, and experimental setup see [54]). In each of the three regions 18 grassland plots were selected such that soil characteristics covered the entire range of environmental conditions and land use regimes present in the Biodiversity Exploratories [54]. A total of 324 phytometers were planted. In May and June 2015, the cover of all vascular plant species in a radius of 15 cm around each phytometer was estimated [54]. No vegetation was removed at the time of transplantation in order to keep disturbance in the transplantation areas as minimal as possible.

After 1 year of in situ growth, phytometer plants were excavated and the rhizosphere was sampled [54]. Since the plants were initially grown axenically, their rhizosphere bacterial communities originated from the Exploratory soils. Soil particles firmly attached to the roots (1–3 mm around the root) were detached by vigorous shaking in sterile 0.5% NaCl solution. Larger soil particles were collected by decanting the supernatant that was filtered through polycarbonate filters (47 mm diameter, 0.2 μm pore size) to also collect smaller, suspended particles. Decanted large particles and filters were combined. Bulk soil was sampled randomly once per plot at a distance of at least 5 cm from one of the phytometer plants and at a depth of ~10 cm. All samples were flash frozen in the field, transported to the laboratory on dry ice, and stored in liquid nitrogen. Of the 324 phytometers planted, 212 were recovered from 50 plots. The remaining were lost due to grazing and four plots were inaccessible at the time of sampling. In parallel, bulk soil samples (one sample per plot) were recovered. To assess the potential effects of phytometer establishment separately, the rhizosphere of 82 naturally occurring individuals of *Plantago lanceolata* and *Dactylis glomerata* were also sampled (Table S1). The resident plant specimens were sampled in the vicinity of the phytometer plants, no further apart than the phytomether plants from one another.

Plant and soil characteristics and climatic data were determined by standard methods (see Supplementary information). The collection of exudates from individual plant specimens was conducted as described earlier [26] and detailed in the Supplementary information (Experimental procedures).

### RNA extraction, cDNA synthesis, library preparation, and sequencing

The goal of the present study was to evaluate the active fraction of the rhizosphere bacterial communities, since these bacteria largely determine the nutrient turnover in this environment. The 16S rRNA was selected as a target molecule. Bacterial RNA was extracted from rhizosphere and bulk soil samples as described by Lueders et al. [55] with appropriate modifications developed for Exploratory soils [56]. After reverse transcription, the V3 region of the 16S rRNA was amplified from the cDNA and processed (see Supplementary information). Sequences were grouped into operational taxonomic units (OTUs) using 99% similarity identity with closed-reference approach, against the SILVA 128 database clustered at 99% similarity [57]. Representative sequences for each OTU were taxonomically assigned using the SILVA 128 database [57]. All Illumina datasets were submitted to the European Nucleotide Archive under the study accession number PRJEB30633.

### Diversity metrics and statistical analyses

Standard diversity variables were determined (see Supplementary information). To quantify the variance in the active rhizosphere bacterial community structure that was explained by the relative abundance of root exudates (Table S2), or the soil and plant variables (Table S3), a variance partitioning analysis was performed (*varpart* function; *vegan* package). Due to collinearity in the root exudate dataset, which arises from a high amount of zeros, these were cropped to exudates present in more than 25 rhizosphere samples. Since soil variables explained most of the variance, a redundancy analysis (RDA) of the correlation between soil variables and the bacterial composition was performed (*rda* function, vegan package). The correlation of the canonical axes with the explanatory matrix was determined with the general permutation test (*anova.cca* function; 999 permutations).

To further quantify the variation in active rhizosphere bacterial communities that was explained by the various variables covered in the present study, a structural equation modelling approach (SEM) was employed on β-diversity data (*sem* function; *lavaan* package). SEM was based on the site scores of the first or additionally the second axis of NMDS for all variables. For bulk soil and active rhizosphere bacterial communities, NMDS were based on weighted UniFrac distances. For plant variables, surrounding plant community, root exudates, soil, and climate variables (Table S3), NMDS was based on Bray–Curtis distances. NMDS based on spatial distance (km) was also included. Data were rescaled as percentages of maximal values. The initial model was simplified by a stepwise removal of uninformative paths, until a suitable model was obtained, indicated by a reduction of the Akaike information criterion. Furthermore, modification indices were used to identify missing paths. The χ^2^ test and the root mean square error of approximation (RMSEA) were used to test the overall goodness of fit. Goodness is indicated by a low χ^2^ and a high probability (*p* > 0.05), and a RMSEA near 0 and a high probability (*p* > 0.05). Because some variables were not normally distributed, these values were computed with Satorra–Bentler correction and we further confirmed the fit of the model using the Bollen–Stine bootstrap test (good fit indicated by high bootstrap *p* and *p* > 0.05; function *bootstrapLavaan*).

To identify the OTUs preferentially associated with the rhizosphere, the differential relative abundances (fold changes) of OTUs between bulk soil and rhizosphere were determined. This was performed individually for each plant species with the *DESeq2* package, using the Wald significance test and the Benjamini–Hochberg *p *value correction. The relative abundance of each rhizosphere-enriched OTU was correlated with the relative abundance of each of the exudate compounds and with soil variables (positive and statistically significant changes, *p* < 0.01). The Pearson correlation (*corr.test* function; *psych* package) was used. To validate the positive and significant correlations between OTUs enriched and root exudates observed, we compared our results with the data available for metabolic traits of cultivated representatives of the OTUs as provided by the Bac*Dive* database [58].

The phylogenetic clustering of OTUs enriched in the rhizosphere was analysed by two independent methods. In a manual approach, monophyletic OTUs were taxonomically identified by mapping onto the full length sequences in the SILVA database. Then the *p*-distances between the aligned full 16S rRNA gene sequences of all OTUs of the same monophyletic cluster were determined with the software MEGA7 and the average value per cluster calculated. In a second approach, the net relatedness index (NRI) and nearest taxon index (NTI) [59] values were calculated using the functions *ses.mpd* and *ses.mntd* of the *picante* package, respectively. Positive values suggest that OTUs are more related to each other than what is predicted by random models, whereby NRI indicates phylogenetic clustering at deep branches and NTI a predominant clustering at the terminal branches. These calculations were limited to the abundant 7744 OTUs (relative abundance > 2 × 10^−4^% [16]).

This work is based on data elaborated by several projects of the Biodiversity Exploratories program (DFG Priority Program 1374). The data publication policy of the Biodiversity Exploratories includes by default an embargo period of three years from the end of data collection/data assembly. These datasets (IDs: 14686, 20489, 20787, and 22366) will be made publicly on the Biodiversity Exploratories Information System (10.17616/R32P9Q) at https://www.bexis.uni-jena.de/PublicData/PublicData.aspx. The information of polar exudates was deposited in the MetaboLights database (https://www.ebi.ac.uk/metabolights/reviewer4dd06f0d-cd80-4e53-a301-e07252a9e778) and is available on request.

## Results and discussion

### Active rhizosphere bacterial communities of phytometers and naturally occurring plants

**Fig. 1:**
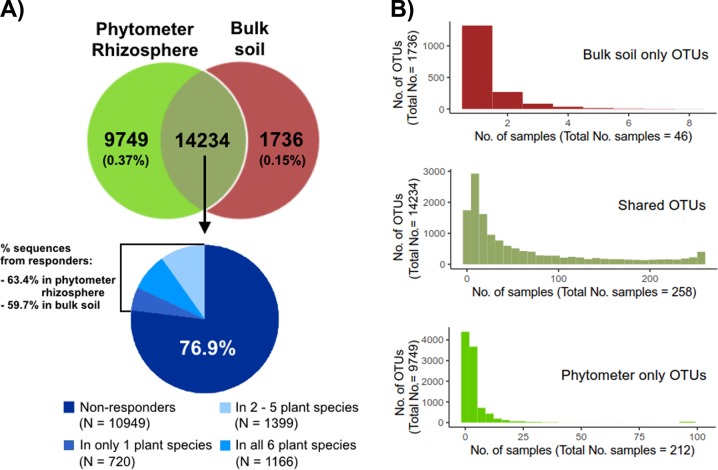
Distribution of OTUs between phytometer rhizosphere and bulk soil compartments. **a** Numbers of unique and shared OTUs and proportion of shared OTUs. From the shared OTUs, 10,949 did not respond to the rhizosphere environment, while 3285 were enriched in the rhizosphere of one to six plant species. Numbers in parentheses and on the left of the bracket give average contribution (relative abundance) of the respective OTUs to all sequence reads in rhizosphere or bulk soil. **b** Frequency of OTUs per sample for bulk soil only, shared or phytometer rhizosphere only OTUs

After quality filtering, denoising, and chimera removal, ∼4·10^8^ 16S rRNA sequences (mean, 1,159,485 per sample) were obtained from the total of 340 samples (212 phytometers, 82 natural plants, and 46 bulk soils). Rarefaction curves nearly reached saturation for the OTUs detected in the rhizosphere, indicating that our sequence inventory covered most of the taxa present in these samples (Fig. S1). Correspondingly, estimates of taxon coverage [60] for individual samples ranged from 98.6 to 99.7%, with an average value of 99.4%. One quarter of the sequences could be taxonomically assigned using the QIIME closed-reference approach against the SILVA SSU Ref 128 database at a sequence similarity level of 99% and were included in the subsequent analysis. After rarefication, sequences were clustered in 27,268 OTUs at 99% sequence identity. Of these, 9749 OTUs (35.7%) were exclusively detected in the phytometer rhizosphere, only 1736 OTUs (6.4%) exclusively in soil, but 14,234 OTUs (52.2%) were present in both (Fig. 1a). However, the OTUs detected exclusively in bulk soil or exclusively in the phytometer rhizosphere were represented by only few (<50) sequence reads and were observed only in one or very few samples (Fig. 1b); they hence constituted a minor fraction of total observations (on average 0.37% of the sequence reads in individual rhizosphere samples). The bacteria that responded (positively or negatively) to the rhizosphere environment were identified for each of the six plant species. A total of 3309 OTUs responded to the rhizosphere environment of at least one plant species by changes in abundance, and the majority (3285) were OTUs shared by rhizosphere and bulk soil (Fig. 1a). A total of 1834 OTUs were enriched in the rhizosphere of at least one plant species, on average constituting 48.1% of all sequence reads in the active rhizosphere bacterial communities. The 1473 rhizosphere-depleted OTUs on average constituted 41.4% of the bulk soil bacterial communities.

A total of 1549 OTUs originated exclusively from natural specimens of *Dactylis glomerata* and *Plantago lanceolata* but occurred only in one or a few samples (data not shown). One year after planting, α-diversity estimates and the composition of active rhizosphere bacterial communities of *Dactylis glomerata* and *Plantago lanceolata* phytometers were similar to naturally occurring individuals (Fig. S2). We therefore conducted all subsequent analyses for phytometer rhizobiomes.

### Active rhizosphere bacterial communities are distinct from, but as diverse as, bulk soil communities

**Fig. 2:**
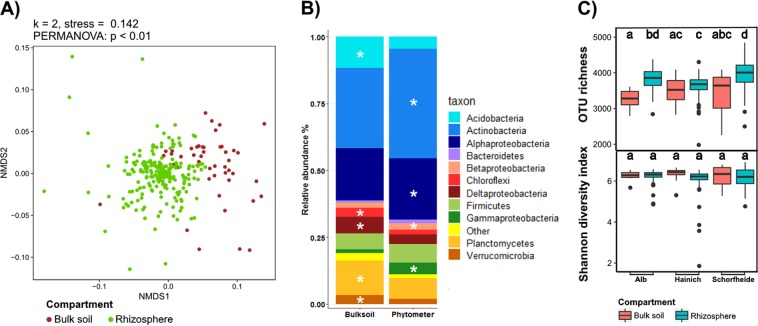
Bacterial community structure and diversity in the rhizosphere of 211 phytometer plants (a single extreme outlier was not included) and corresponding bulk soil (46 samples). **a** NMDS plot of bacterial composition based on weighted UniFrac distances at OTU level, coloured by compartment. **b** Average relative abundances of bacterial phyla and proteobacterial classes. Only phyla and proteobacterial classes with average relative abundances of >1% are shown. Taxa with significantly higher relative abundances present in the rhizosphere or bulk soil (*t*-test, *p* < 0.01) are marked with asterisks. Rhizosphere bacterial communities of the six phytometer plant species were significantly enriched for *Actinobacteria* (mean of 40.9 versus 30.1%), *Alphaproteobacteria* (23.1 versus 19.3%), *Bacteroidetes* (1.6 versus 0.9%), and *Gammaproteobacteria* (4.1 versus 1.4%). On the opposite, the rhizosphere was significantly depleted for *Acidobacteria* (4.6 versus 11.7 %), *Planctomycetes* (7.6 versus 13%), *Chloroflexi* (1.9 versus 3.4%), *Deltaproteobacteria* (3.6 versus 6%), and *Verrucomicrobia* (2 versus 3.3%). **c** Richness and diversity estimates at OTU level for rhizosphere and bulk soil bacterial communities in the three exploratory regions. Letters on top of each boxplot denote statistically significant differences between all exploratory × compartment combination (*p* < 0.01, multcomp test)

The overall composition of active rhizosphere bacterial communities of phytometers differed significantly from those in bulk soil on the OTU level (Fig. 2a) as well as on the phylum/class level (Fig. 2b). Active rhizosphere bacterial communities of phytometers grown in Schwäbische Alb and Schorfheide grasslands consisted of ∼4000 OTUs and were significantly richer than those of the surrounding bulk soils. Shannon diversity indices did not differ between rhizosphere or bulk soil samples or between the three regions. However, the evenness of rhizosphere communities in the Alb and Hainich grasslands was slightly, but significantly, decreased compared with the corresponding bulk soil communities (Fig. 2c).

By comparison, a reduced bacterial OTU diversity and richness has been reported for the rhizosphere of several other plant species [16, 18, 32] although contradictory results exist, sometimes even for the same plant species [2, 15, 61]. The decrease in OTU richness in the rhizosphere has been attributed to a homogenizing effect of rhizosphere processes that reduce niche dimensions [16]. Nevertheless, we did not observe a reduction of active rhizosphere bacterial diversity across the different soil types and under the environmental conditions tested. Possible reasons for the discrepancies to some of the previous DNA-based studies are the presence of taxa that consist mostly of dormant bacteria and/or the presence of long-lived relic bacterial DNA [62]. Both are likely to be more frequent in substrate-limited bulk soil than in the rhizosphere and thereby could lead to higher DNA-based estimates for the α-diversity and species richness in bulk soil. This reflects the importance of examining the active fraction of bacteria when investigating environments that differ in substrate availability such as rhizosphere and bulk soil. Based on our results, the rhizosphere provides as many niche opportunities for bacterial communities as the surrounding bulk soil.

A total of 50 bacterial phyla were identified in the present study. Median values for the relative abundance of representatives of these phyla were 0.0046 and 0.0015% for bulk soil and rhizosphere, respectively, but eight phyla, and within the phylum *Proteobacteria*, four classes (*Alpha-*, *Beta-*, *Gamma-*, *Deltaproteobacteria*), surpassed a median relative abundance of 1% (Fig. 2b). Overall, these eight most abundant phyla accounted for 98.5% of OTUs detected in the phytometer rhizosphere and for 97.3% of OTUs in the bulk soil. Active rhizosphere bacterial communities of the six phytometer plant species were significantly enriched for *Actinobacteria*, *Alphaproteobacteria*, *Bacteroidetes,* and *Gammaproteobacteria*, but depleted for *Acidobacteria*, *Planctomycetes*, *Chloroflexi*, *Deltaproteobacteria*, and *Verrucomicrobia* (Fig. 2b). An increased abundance of *Alphaproteobacteria*, *Gammaproteobacteria*, and *Bacteroidetes* has been described for the rhizospheres of cultivated plants like maize [18], lettuce [35], and grape [32], but occasionally also for non-cultivated plants like grasses (*Avena fatua*; [15, 16, 38]). According to our results, this general trend is also true for the active rhizosphere bacterial communities of six native plant species of central European grasslands. Similarly, previous studies reported a decreased abundance of *Acidobacteria*, *Chloroflexi*, *Planctomycetes*, and *Verrucomicrobia*. However, the significant enrichment of active *Actinobacteria* in the rhizosphere of the six plant species has previously not been observed [16, 38].

The 3309 OTUs responding by changes in abundance (Fig. S3) were analyzed in more detail. Within each bacterial phylum or class, 3–38% of all OTUs responded to the rhizosphere (Fig. S3A). Positively responding OTUs dominated in the *Actinobacteria*, *Alphaproteobacteria*, *Gammaproteobacteria*, and *Bacteroidetes*, but also in *Betaproteobacteria*, *Firmicutes*, and *Cyanobacteria* (Fig. S3B, C). Ten phyla and classes, such as the *Deltaproteobacteria*, *Acidobacteria*, *Chloroflexi*, *Gemmatimonadetes*, *Planctomycetes*, and *Verrucomicrobia*, encompassed a larger fraction of negative responders.

Two *Streptomyces* OTUs were the most abundant active members of the rhizosphere and on average amounted to 1.57 and 1.35% of the total active rhizosphere bacterial community (Tables S4 and S5). Only 18.3% of the positively responding OTUs were affiliated to 28 genera, which were previously known for their association with plant roots and their role as plant growth promoting bacteria [63, 64], including *Streptomyces*, *Pseudomonas*, *Mesorhizobium*, and *Rhizobium*. However, the remaining 81.7% (1560) of OTUs were unclassified or belonged to 259 genera, which so far have not been explored for their possible beneficial interaction with plant roots. These bacteria include members of, e.g. *Sphingomonas*, *Cellulomonas,* and *Aeromicrobium*, which have been previously described as capable of phosphate solubilisation [65, 66] and siderophore production [67]. Our study thus identified a considerable number of bacterial sequence types that represent potential candidates for future studies of plant-growth promotion or phytopathogenic relationships in natural grasslands. In this respect, the actinobacterial genera *Streptomyces* and *Nocardioides*, and among the former the two strongly responding sequence types (AJ316140.1.1445 and JF167768.1.1343) are of particular interest.

### Specific responses of bacterial OTUs to defined root exudates

Bacterial adaptations to the plant rhizosphere environment include motility, chemotaxis, quorum sensing, lipopolysaccharide synthesis [34], increased biofilm formation, horizontal gene transfer [4], or particular substrate utilization profiles [30]. Of these, taxon-specific responses to individual root exuded compounds could constitute an important driver of the bacterial community assembly observed in the rhizosphere. Therefore we analyzed the interdependence between the abundance of the 1834 positively responding rhizobacterial OTUs and the relative abundance of individual polar compounds of root exudates, which are mainly part of the primary metabolism.

**Fig. 3:**
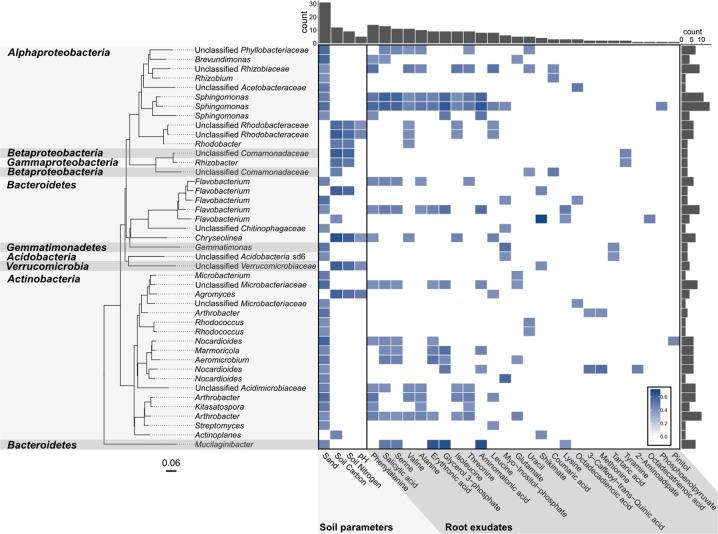
Heatmap of statistically significant (*p* > 0.01, Pearson correlation) and positive correlations between rhizosphere-enriched OTUs and soil variables and known metabolites; with associated phylogenetic tree from SILVA SSU 128, cropped to the desired OTUs. For simplicity only OTUs that were correlated with both soil variables and root exudates with *R*^2^ > 0.4 are shown. Extended heatmaps are shown in Figs. S4 and S5 for soil variables and root exudate compounds, respectively. Each tip represents a single OTU labelled according to the genus name. Phyla and proteobacterial classes are indicated to the left in the shaded areas. Histogram on the top shows the number of correlations registered for each variable and, conversely, the histogram on the right depicts the number of correlation observed for each OTU

Statistically significant and positive correlations with at least one exudate compound could be detected for 1345 of the OTUs (73.3%), of which 655 OTUs were associated with chemically identified compounds (Fig. 3; Fig. S4). By comparison, significant and positive correlations with at least one soil parameter were found for 969 (52.8%) of the OTUs (Fig. 3; Fig. S4), particularly with the relative amount of total nitrogen (329 OTUs). Potentially, some of the remaining responders that were not correlated to the polar exuded metabolites may be adapted to other rhizodeposits like members of the *Methylobacteriaceae*, which are reported to utilize volatile plant organic compounds, especially methanol [68, 69]. Indeed, 10 OTUs of this family were detected in our study but were not correlated to any of the identified root exuded compounds. This is also the case for the 45 OTUs of *Planctomycetes*, a phylum with potential for methylotrophy [70]. In addition, at least some of the rhizosphere OTUs identified are likely to respond to the production of secondary metabolites and plant polymers not covered by the present study [71, 72].

Most of the positive associations observed with exuded metabolites were with amino acids and organic acids, especially with valine, isoleucine, leucine, phenylalanine, salicylic acid, and serine (162, 154, 151, 146, 143, and 141 positive correlations, respectively) (Fig. 3; Fig. S5). These compounds are likely exploited as a nutrient source [73]. In fact, 18.7% of these positive correlations could be substantiated by comparison to phenotypic characteristics of closely related strains of the same genus as provided by the BacDive database [74]. One of the strongest relationships detected was between an OTU belonging to the genus *Aeromonas* and coumaric acid (Fig. S5). This is consistent with the capacity for coumaric acid utilization by several members of this genus, such as *A. hydrophila*, *A. sobria,* and *A. caviae* [75]. Furthermore, *Sphingobium paulinellae*, *algicola*, and *scionense* [76, 77] utilize N-acetylglucosamine and *Massilia flava* has tryptophane deaminase activity [78]. The congruence with known physiological traits of described genera indicates that our combined approach can also provide information on the substrate adaptations of not-yet-cultured rhizosphere bacteria.

To investigate the phylogenetic coherence of rhizosphere associated bacteria, the sizes of all monophyletic clusters of positively responding OTUs were determined (Fig. S6A) and similarity values within the clusters detected were calculated (Fig. S6B). The vast majority (1590) of rhizosphere-enriched OTUs constituted single lineages, while 206 formed monophyletic groups with one other rhizosphere-responsive OTU, 11 clusters encompassed three OTUs, and only one a total of five OTUs (Fig. S6A). The average similarity within sequence clusters was 97.6% and the median 98%, whereas only four values ranged below the similarity value for genera (Fig. S6B). In addition, the NRI and NTI values were calculated, since they quantify overall clustering of taxa on a tree [59]. Rhizosphere-enriched bacterial OTUs were found to be phylogenetically overdispersed (NRI of −3.01, *p* = 0.998) over the entire tree, and phylogenetic clustering was found mainly towards terminal branches (NTI of 13.85). This supports the conclusion that traits for rhizosphere competence are not evolutionarily conserved over large phylogenetic distances within higher taxa and that the adaptation to the rhizosphere likely represents a convergent bacterial trait, contrary to some previous suggestions [16, 38]. Horizontal gene transfer may contribute toward the acquisition of rhizosphere competency since genes involved in HGT occur at a higher abundance in the rhizosphere than in bulk soil [4].

### Rhizobiome diversity is linked to the abiotic and biotic environment

Significant differences in the OTU richness of active rhizosphere bacterial communities were observed when soil types and plant species were analysed separately (Fig. S7A, B). Active rhizosphere bacterial communities of grasses were richer than those of forbs. Considering the Shannon diversity index, however, no differences were observed between active rhizosphere bacterial communities of different plant species, but between those in different soils. In particular, the diversity estimate for histosols was significantly higher than for any other soil type (Fig. S7B). The same pattern was also observed for bulk soil (data not shown), which showed that the specific conditions in histosols, like the high organic matter content and the presence of anoxic habitats under waterlogged conditions [79], may provide additional niches for bacteria (e.g. anaerobes) and result in a higher diversity also in the rhizosphere. Evenness did not differ between plant species, but between soil types. Again, values for histosols were significantly higher than for other soil types. Significant differences between leptosols and luvisols were also observed. Overall, soil type had a stronger influence on diversity and evenness of active rhizosphere bacterial communities, while both plant species and soil type were of similar importance for OTU richness (Fig. S7C).

**Fig. 4:**
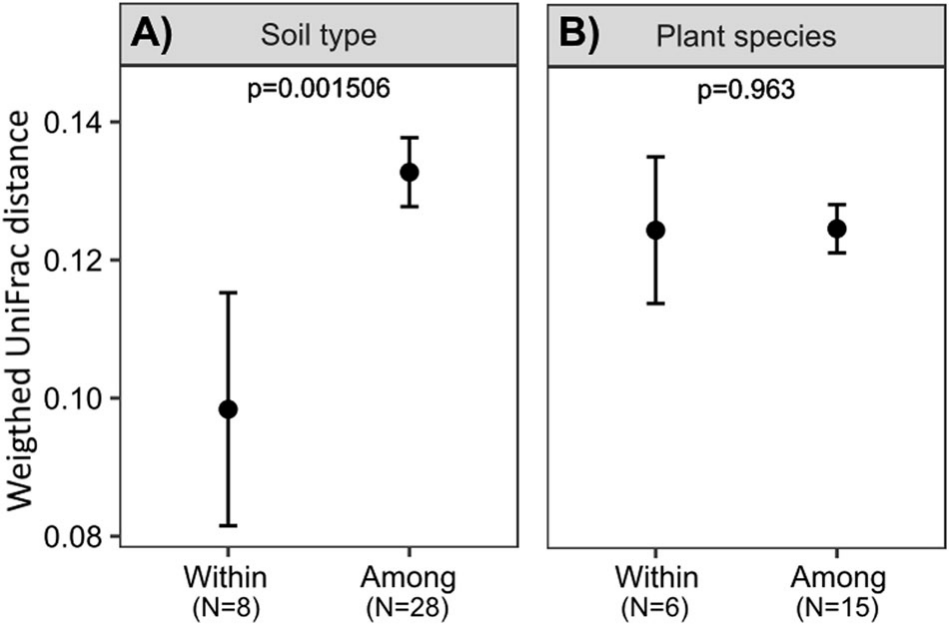
Influence of **a** soil type and **b** plant species on the similarity of rhizosphere bacterial communities. Pairwise comparisons of weighted UniFrac distances were conducted among the 212 rhizosphere communities, either those in the same soil type or associated with the same plant species (category “Within”) or those in two different soil types or associated with two different plant species (category “Among”). Black circles reflect means for all pairwise comparisons within each of the eight soil types, for all combinations of two out of eight soil types (*n* = 28), within the six plant species, and for all combinations of two out of six plant species (*n* = 15) *p* values were determined through *t*-tests

Soil type had an even more pronounced effect on community dissimilarity (β-diversity) between rhizosphere communities, since values of the weighted UniFrac distances between rhizosphere communities of different plant species in the same soil type were significantly lower than values between active rhizosphere bacterial communities in different soil types (Fig. 4a). In fact, rhizobiomes of the same plant species growing in different soil types were as divergent as those of all different plant species (*p* = 0.963) (Fig. 4b). When analyzed separately for each soil type, the β-diversity of rhizosphere communities was similar within the same plant species and between different plant species in five of the six soil types. A lower β-diversity between bacterial communities of the same plant species was only observed for Luvisols, albeit of limited significance (Fig. S8).

In addition, the active rhizosphere bacterial communities differed among the three Biodiversity Exploratories regions (Fig. S9A). Compared with Hainich and Schorfheide-Chorin, samples from the Schwäbische Alb had a higher proportion of *Alphaproteobacteria*, *Deltaproteobacteria*, *Planctomycetes*, and *Verrucomicrobia*. Conversely, the samples from Hainich harboured more *Actinobacteria* and the samples from Schorfheide-Chorin were enriched with *Firmicutes* and *Bacteroidetes* (Fig. S9B). These differences between exploratory regions might be due to spatial separation of bacterial communities and/or due to systematic differences in bedrocks and pedogenesis, or regional climate. Because (i) differences in soil characteristics were also positively correlated to geographic distance (Mantel statistic *r* = 0.22, *p* < 0.01), (ii) seven of the eight soil types studied occur only in a single region of the Exploratories (Table S1), and (iii) physicochemical conditions differed considerably between many of the soil types (Fig. S10A), the effect of geographic distance is confounded with the distinct distribution of soil types and different climate in the three regions. An analysis by a linear model confirmed that the effect of the specific geographic distribution of soil characteristics on the structure of active rhizosphere bacterial communities was larger than that of geographic distance itself (Fig. S10B).

### Potential drivers of the variation in grassland rhizobiomes

**Fig. 5:**
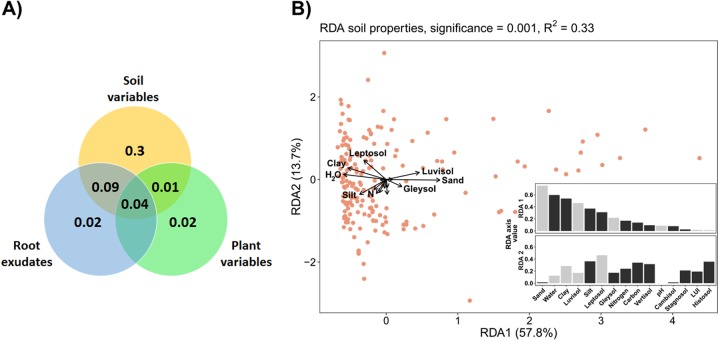
Variance of active rhizosphere bacterial communities at OTU level. **a** Variance partitioning analysis depicting the proportions of variance explained by the ten plant variables, ten soil variables (Table S3), and 148 root exudates (Table S2, after removal of the exudates that were present in only ≤25 samples to account for collinearity, see ‘Material and methods' section). **b** Redundancy analysis (RDA) of the relationship between soil variables (constraining variable) and the weighted Unifrac distances between bacterial communities. Dots indicate individual rhizosphere samples. The arrow lengths and directions correspond to the variance that can be explained by the individual soil variables; only the eight variables with the highest absolute loadings (RDA1) are named. Insert: loadings (RDA axis values) for the individual soil variables. Grey and black colours of the bars indicate positive and negative values, respectively

It has been suggested that environmental conditions may affect the plant rhizobiome indirectly through determining the developmental stage and physiological status of the host plant and thereby the composition of root exudates. Alternatively, specific soil physicochemical conditions may directly select for particular bacterial species in the rhizosphere [39]. When the quantitative contribution of all variables covered by the present study (Tables S2 and S3) to differences in the rhizobiome composition was assessed by variance partitioning, soil characteristics alone were found to explain most of the variance (30%). Synergistic effects of polar root exudates and soil characteristics explained another 9%, whereas other plant characteristics had little effect (Fig. 5a). Phytometer plant individuals underwent different development depending on the physicochemical conditions and surrounding vegetation on the grassland fields. Therefore, plant biomass was used to evaluate the overall status of the plants’ performance [80] in a variance partitioning analysis, but its effect on rhizosphere bacterial communities was negligible and the effect on polar root exudate composition very small (3%) (further data not shown). Among soil characteristics, soil texture, water content, and soil type contributed most to explain rhizobiome composition (Fig. 5b). While it is known that physicochemical soil conditions have a stronger effect than land use on bulk soil bacterial communities [81], our results indicate that this is also the fact for the rhizobiome.

**Fig. 6:**
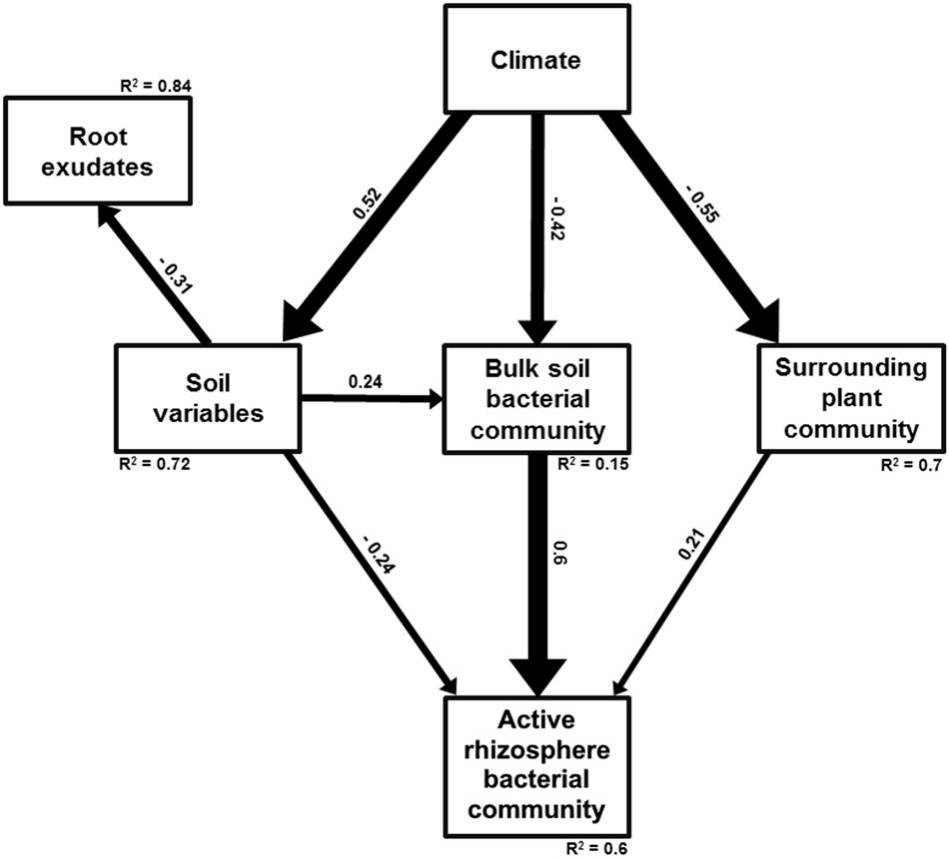
Structural equation modelling evidencing significant relationships (*p* < 0.01) between differences in soil variables, surrounding plant community, climate, root exudate composition, and their influence on differences in bulk soil and rhizosphere bacterial communities. Host plant variables were tested but are not displayed as no relationship involving these was statistically significant. The thickness of the arrows indicates the strength of the causal relationship, supplemented by a path coefficient. *R*^2^ values denote the amount of variance explained by the model for the response variables. Our overall SEM model fit was satisfactory (χ2 = 3.71, *p* = 0.59; RMSEA = 0.000, Bootstrap *p* = 0.48)

We assessed the potential drivers of the observed changes in rhizosphere and bulk soil bacterial communities in more detail, employing a SEM approach. The model that yielded the best fit for the scores of the first NMDS axis (Fig. 6) revealed that, of all variables assessed, changes in the composition of the bulk soil bacterial communities constituted the strongest direct driver of changes in the composition of rhizosphere communities of the six model plant species. The composition of surrounding plant communities and soil variables exerted additional, weaker effects. Soil variables had an additional, indirect effect on the rhizobiome due to their influence on the composition of the bulk soil bacterial communities. Notably, soil variables determined the composition of root exudates after the plant phytometers were retrieved form the soil. In contrast, no link between the composition of root exudates and of active rhizosphere bacterial communities could be detected (Fig. 6). Extending the SEM analysis to scores of the second NMDS axis (Fig. S11) identified some additional, but weak links between the composition of surrounding plant communities and root exudates, but still did not show an effect of root exudate composition on active rhizosphere bacterial communities. Contrary to plants growing separately in mesocosms or in monoculture [20, 21, 65, 66] and in a similar soil type [16, 38], the specific traits of host plants growing in the complex exploratory grasslands had no discernible effect on the composition of their own active rhizobiome.

Whereas some exuded compounds can cause distinct changes in rhizosphere bacterial communities of particular model plant species when grown separately [82], such a direct effect of root exudates on the rhizobiome maybe masked in complex grasslands because of interactions between the rhizospheres of different plant species via polar exudates [83] and/or the selective effect of species-specific litter originating from surrounding plants that is degraded in the rhizosphere when primed by root exudates [31, 84]. It is argued that the effect of root exudates on rhizobiome assembly is rather limited by the spatiotemporally confined exudation that mostly occurs at root apices, by the nonspecific chemical nature of the exuded molecules that also occur in other rhizodeposits, and by the rapid modification of exudates by some rhizosphere bacteria [6].

Instead, our data suggests that assembly of active rhizosphere bacterial communities in temperate grasslands is strongly, and almost exclusively, controlled by recruitment from bulk soil bacterial communities. Microbial community assembly in bulk soil, in turn, is affected by physicochemical soil characteristics, confirming earlier findings [72]. On a local scale and for similar soils, plant community composition in grasslands has been found to be a predictor of bulk soil microbial communities [85], possibly due to differences in the quality, quantity, and diversity of the litter that is produced by different plant communities and that is used as major growth substrate of bacteria in bulk soil [86]. However, over the larger distances, different climatic regions, and the broad range of soil types covered by the present study, the β-diversity of bulk soil bacterial communities was largely affected by abiotic variables rather than by changes in surrounding plant communities. The habitat conditions in the rhizosphere, including the presence of root exudates, select for distinct rhizobiomes (Fig. 2a), but these habitat conditions do not seem to be specific for individual plant species in dense grasslands, resulting in a rhizobiome community assembly that is largely uncoupled from the species identity of the host plant.

## Conclusions

Understanding the controls of nutrient cycling, productivity, disease risk, and symbiotic interactions of grasslands provide the basis for improvements in their management [8, 72] but requires prior and in-depth knowledge of the determinants of microbial community dynamics and composition [42]. Our phytometer study of 27,268 bacterial OTUs identified the recruitment process from bulk soil communities as the major direct driver of the composition of active rhizosphere bacterial communities in species-rich natural grassland ecosystems. A rhizobiome community assembly that is largely uncoupled from the species identity of the host plant has previously been reported for two arcto-alpine herb species [61] and for different members of plant communities in vineyards [30]. Based on our large-scale study, this process also occurs in temperate grasslands and hence in one of the dominant ecosystem types. Whether and to which extent it effects plant-soil feedbacks and the increase of ecosystem productivity with plant species diversity (the ‘overproductivity'; [9]) warrants further study. Our study also identified numerous previously unknown taxa that are involved in rhizosphere dynamics and hence represent suitable targets for future manipulations of the plant rhizobiome. The significant association of individual OTUs with specific root exuded compounds provide first information on their substrate utilization and thus a handle for future cultivation attempts and application as rhizosphere inoculants.

## Supplementary information

Supplementary Information - Drivers of the composition of active rhizosphere bacterial communities in temperate grasslands
