# Supplementary material for: Drivers of the composition of active rhizosphere bacterial communities in temperate grasslands
Source: ISME J. 2019 Oct 28;14(2):463–75. doi: 10.1038/s41396-019-0543-4 (PMC6976627; doi:10.1038/s41396-019-0543-4)
Supplement: Supplementary file 1 — Supplementary Information - Drivers of the composition of active rhizosphere bacterial communities in temperate grasslands [file 41396_2019_543_MOESM1_ESM.docx]

**Supplementary Information**

**Drivers of the composition of active rhizosphere bacterial communities in temperate grasslands**

Selma Vieira^1^, Johannes Sikorski^1^, Sophie Dietz^2^, Katharina Herz^3^, Marion Schrumpf^4^, Helge Bruelheide^3,5^, Dierk Scheel^2,5^, Michael W. Friedrich^6^, Jörg Overmann^1,7^*

^1^ Leibniz Institute DSMZ-German Collection of Microorganisms and Cell Cultures, Inhoffenstraße 7B, 38124 Braunschweig, Germany

^2^ Leibniz Institute of Plant Biochemistry, Weinberg 3, 06120 Halle (Saale), Germany

^3^ Martin Luther University Halle-Wittenberg, Institute of Biology / Geobotany and Botanical Garden, Am Kirchtor 1, 06108 Halle (Saale), Germany

^4^ Max Planck Institute for Biogeochemistry, Hans-Knöll-Straße 10, 07745 Jena, Germany

^5^ German Centre for Integrative Biodiversity Research (iDiv) Halle-Jena-Leipzig, Deutscher Platz 5e, 04103 Leipzig, Germany

^6^ Microbial Ecophysiology group, Faculty of Biology/Chemistry & Center for Marine Environmental Science (MARUM), University of Bremen, Leobener Straße 3, 28359 Bremen, Germany

^7^ Braunschweig University of Technology, Universitätsplatz 2, 38106 Braunschweig, Germany

* Correspondence: J. Overmann, Leibniz Institute DSMZ – German Collection of Microorganisms and Cell Cultures, Inhoffenstrasse 7B, 38124 Braunschweig, Germany.

Tel. ++49-531-2616-352. Fax ++49-531-2616-418. E-mail: Joerg.Overmann@dsmz.de

**Experimental procedures**

*Determination of plant and soil characteristics, and climatic data*

After collection, plants were weighed both fresh and after drying [1]. After grinding roots to fine powder, root nitrogen and carbon contents were determined by dry combustion using an elemental analyzer (vario EL cube; Elementar, Hanau, Germany) [1]. For each plot, soil pH, carbon and nitrogen content were measured. Soil texture data (percentage of sand (2 – 0.063 mm), silt (0.063 – 0.002 mm) and clay (< 0.002 mm)) was determined previously for the same sites [2]. Land-use intensity (LUI) was calculated for the year 2014 employing the index established earlier [3]. Each plot was equipped with a microclimate station to measure air temperature at 10 and 200 cm above ground, relative humidity at 200 cm above ground, and soil moisture at 10 cm depth. We calculated mean values of the four parameters for the entire experimental period from May 2014 to Jul 2015 using the monthly means [1, 4]. Precipitation data was extracted from RADOLAN RW products (DWD Climate Data Center (CDC); www.dwd.de/RADOLAN).

*Collection, extraction, measurement and analysis of root exudates*

Briefly, exudates were collected by a field exudate collection method [5, 6]. After extracting the rhizosphere soil by washing the roots in 0.5 % sodium chloride solution (NaCl) for 10 min, the roots were washed with deionised water. Then, the complete roots of the intact plant were placed in 250 ml brown plastic vessels containing 200 ml of deionised water of HPLC-Grade for 2 hours. Water samples without plants served as extraction controls. Ribitol (10 µM) was added to each solution as internal standard. Samples were stored at -20°C in the field and until further processing. 200 µL of each sample were derivatized and metabolites were measured with a gas chromatograph coupled to an electron impact ionisation mass spectrometer (GC-MS) according to Herz *et al*. [5]. An untargeted metabolite profiling approach was used to annotate the exuded compounds. Metabolites were identified as detailed previously [5]. Classification of metabolites was done according to their affiliation to natural substance classes. All metabolites and compounds that were detected in ≥ 50% of the controls were eliminated from the analysis. The remaining substances in each sample were normalized to the internal ribitol standard.

*RNA extraction, cDNA synthesis, library preparation and sequencing*

Samples were thawed on ice and transferred to a 2 ml screw cap tube containing 0.7 g of sterilized zirconium/silica beads (diameter, 0.1 mm), 750 µl sodium phosphate solution (112.9 mM Na_2_HPO_4_, 7.1 mM NaH_2_PO_4_) and 250 µl TNS-Buffer (500 mM Tris-HCl pH 8, 100 mM NaCl, 10% sodium dodecyl sulfate). Cells were disrupted by bead-beating (2 times at 6.5 m·s^-1^ for 45 s). After centrifugation, samples were extracted with phenol-chloroform-isoamyl alcohol (25:24:1 v/v/v), then chloroform-isoamyl alcohol (24:1), and nucleic acids were pelleted by the addition of polyethylene glycol and centrifugation. Pellets were washed with cold ethanol (70%) and resuspended in 20 to 50 µl Tris-HCl buffer (pH 8.5). RNA was prepared by digestion of co-extracted DNA with RNase free DNase I (ThermoScientific, Waltham, MA, U.S.) according to the instructions of the manufacturer, and subsequently precipitated in sodium acetate (3M, pH 5.2) and isopropanol (99.5%), washed with ethanol (70%, v/v), and resuspended in RNase free water. Concentrations of RNA were determined using the Quant-iT RiboGreen RNA Assay Kit (Life Technologies, Darmstadt, Germany) and a microtiter plate reader (Tecan Inﬁnite 200 PRO; Männedorf, Switzerland). RNA extracts were treated with RiboLock RNase inhibitor (final concentration 1 U µl^-1^; Fermentas, Waltham, MA, USA) prior to reverse transcription PCR. For synthesis of cDNA from extracted RNA, the GoScript Reverse Transcription System was employed according to the protocol of the manufacturer (Promega, Madison, WI, USA) using random hexamers.

The V3 region of the 16S rRNA was amplified using modified primer pairs 341F (5’-CCTACGGGWGGCWGCAG-3’) and 518R (5’-CCGCGGCTGCTGGCAC-3’) [7] which contained Illumina adapter sequences and binding sites for sequencing primers. Additionally, the reverse primer included an index region of 6 nucleotides [8]. All samples were amplified in triplicates. The reaction mix (final volume of 50 µl) contained 10 µl PCR buffer (5x; GC Phusion buffer), 1 µl dNTP mix (10 mM each), 0.2 µl each of forward and reverse primers (50 µM each), 1.5 µl dimethyl sulfoxide (DMSO; 100% v/v), and 1 µl Phusion High-Fidelity DNA Polymerase (2 U µl^-1^; Thermo Scientific, Waltham, USA). Amplification proceeded by an initial denaturation step at 94°C for 5 min, followed by 20 cycles at 94°C for 15 seconds, 59°C for 15 seconds, 72°C for 15 seconds, and final extension step at 72°C for 7 minutes. Amplifications were carried out in a Veriti 96-well thermal cycler (Applied Biosystems, Foster city, CA, USA). Amplicons were purified in 2% Metaphor (Lonza group, Basel, Switzerland) agarose gels to allow separation of products from primers and primer dimers. Subsequently, the PCR products were cleaned with NucleoSpin Gel and PCR Clean-up Kit (Macherey-Nagel, Düren, Germany) and quantified using Qubit® dsDNA HS Assay Kit (Life Technologies, Carlsbad, CA, US). Quality was checked with a 2100 Bioanalyzer (Agilent Technologies, Santa Clara, CA, U.S.) and samples were pooled in equal amounts. Sequencing was performed on a HiSeq 2500 (Illumina, San Diego, CA, USA) in a paired-end run, yielding a total of 4.70 x 10^8^ sequence reads of 100 bp length.

Reads were assigned to the samples using the index sequence of 6 bp. Downstream processing included the trimming to 100 bp and the removal of primer dimers and adapters, employing detection methods implemented in FastQC (http://www.bioinformatics.babraham.ac.uk/projects/fastqc/). The remaining reads were joined using fastq-join [9] and subsequently checked for chimeric sequences with UCHIME (Usearch 5.2.32 10) applying the GOLD database from ChimeraSlayer (http://drive5.com/otupipe/gold.tz) as reference. Data were analysed using QIIME 1.9.1 [10].

*Diversity metrics and statistical analyses*

All analyses were performed using the R program, version 3.5 [11]. Sequences were rarified to the lowest number of sequences obtained per sample (i.e., 102 311) using the R package *phyloseq*. This package was also used for the determination of relative abundances of taxa, of α-diversity and β-diversity estimates for rhizosphere and bulk soil bacterial communities. The individual effects of soil type and plant species on the α-diversity metrics were determined using a linear model (function *lm*; *stats* package). The subsequent ANOVA analysis (type II) was performed using the *Anova* function from the *car* package. The diversity estimates were based on weighted UniFrac distances. Rarefaction analysis was performed using the *iNEXT* package in R. Multivariable analysis results were visualized by Non-Metric Multidimensional Scaling (NMDS). Variations in bacterial community composition that were explained by soil compartment (bulk or rhizosphere) or plant growth conditions (natural vs phytometer) were tested for significance using permutational analysis of variance (*adonis* function, *vegan* package, 999 randomizations). To determine significant differences in relative abundances or in α-diversity metrics between multiple groups (N > 2), a multiple comparison analysis of variance was done (Tukey’s all pair comparisons), with the package *multcomp* (function *glht*). For comparisons between two groups, the t-test was employed.

The relationship between spatial distance (km) and soil variables (Bray-Curtis distance) was evaluated with a Mantel test using the function *mantel* from the *vegan* package. The individual effects of these parameters on the composition of rhizosphere bacterial communities (weighted UniFrac distances) were determined using a linear model calculated with the function *lm* from the *stats* package. The subsequent ANOVA analysis (type II) was performed using the *Anova* function from the *car* package. The principal coordinate analysis of soil properties, based on Bray-Curtis distances, was done using the function *cmdscale* of the vegan package.

The trees and heatmaps showing the correlation between enriched OTUs and root exudates or soil parameters were obtained employing the *ggtree* package. The heatmap depicting mean relative abundance of exudates of distinct plants was obtained with the *gplots* package. All other plots were generated using the R package *ggplot2*.

**References**

1. Herz K, Dietz S, Haider S, Jandt U, Scheel D, Bruelheide H. Drivers of intraspecific trait variation of grass and forb species in German meadows and pastures. *J Veg Sci* 2017; **28**: 705–716.

2. Solly EF, Schöning I, Boch S, Kandeler E, Marhan S, Michalzik B, et al. Factors controlling decomposition rates of fine root litter in temperate forests and grasslands. *Plant Soil* 2014; **382**: 203–218.

3. Blüthgen N, Dormann CF, Prati D, Klaus VH, Kleinebecker T, Hölzel N, et al. A quantitative index of land-use intensity in grasslands: Integrating mowing, grazing and fertilization. *Basic Appl Ecol* 2012; **13**: 207–220.

4. Bruelheide H. Interpolated climate data of BELOW subplots, 2014 - 2015 v1.0.0. *Biodivers Explor Inf Syst Dataset https//www.bexis.uni-jena.de/* 2018; Dataset Id=22366.

5. Herz K, Dietz S, Gorzolka K, Haider S, Jandt U, Scheel D, et al. Linking root exudates to functional plant traits. *PLoS One* 2018; **13**: 1–14.

6. Dietz S, Herz K, Döll S, Haider S, Jandt U, Bruelheide H, et al. Semi-polar root exudates in natural grassland communities. *Ecol Evol* 2019; (accepted).

7. Muyzer G, De Waal EC, Uitterlinden AG. Profiling of complex microbial populations by denaturing gradient gel electrophoresis analysis of polymerase chain reaction-amplified genes coding for 16S rRNA. *Appl Environ Microbiol* 1993; **59**: 695–700.

8. Bartram AK, Lynch MDJ, Stearns JC, Moreno-Hagelsieb G, Neufeld JD. Generation of multimillion-sequence 16S rRNA gene libraries from complex microbial communities by assembling paired-end Illumina reads. *Appl Environ Microbiol* 2011; **77**: 3846–3852.

9. Aronesty E. Comparison of sequencing utility programs. *Open Bioinforma J* 2013; **7**: 1–8.

10. Caporaso JG, Kuczynski J, Stombaugh J, Bittinger K, Bushman FD, Costello EK, et al. QIIME allows analysis of high-throughput community sequencing data. *Nat Methods* 2010; **7**: 335–336.

11. R Core Team. R: a language and environment for statistical computing. *R Found Stat Comput Vienna, Austria* . 2018.

12. Yarza P, Yilmaz P, Pruesse E, Glöckner FO, Ludwig W, Schleifer K-H, et al. Uniting the classification of cultured and uncultured bacteria and archaea using 16S rRNA gene sequences. *Nat Rev Microbiol* 2014; **12**: 635–645.

**Supplementary Figures**


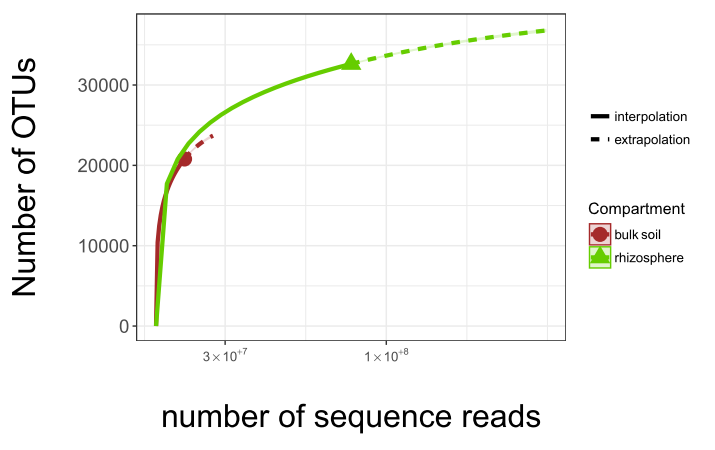


**Figure S1** Rarefaction curves for all rhizosphere and bulk soil samples.

**
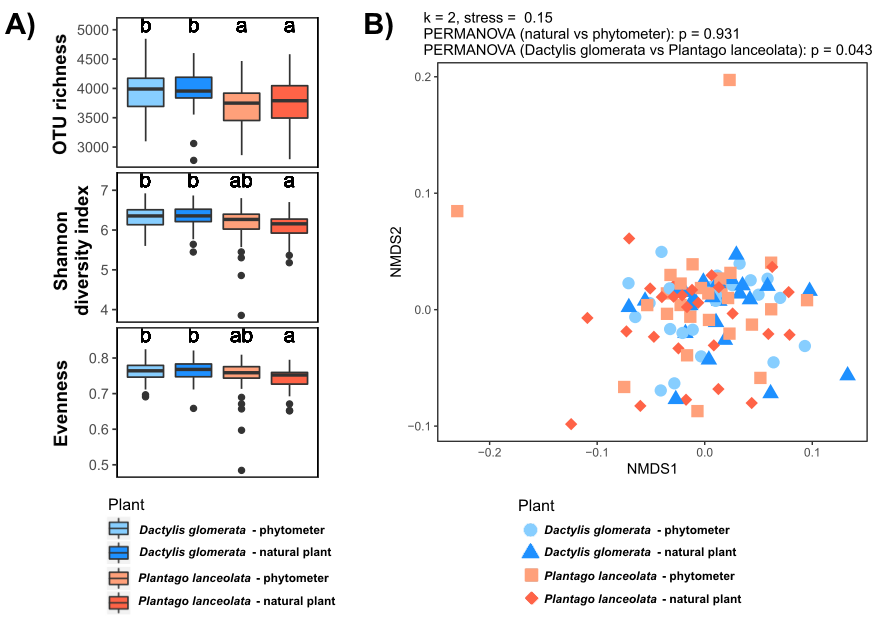
**

**Figure S2** – Comparison of rhizosphere community structure of natural and phytometer plant individuals of *Dactylis glomerata* and *Plantago lanceolata*. **A)** Alpha diversity measures for bacterial communities at OTU level. Letters denote significant differences (p < 0.01) as indicated by the multcomp test. **B)** NMDS plot comparing the bacterial composition of the rhizospheres of natural occurring specimens and phytometer plants of both plant species based on weighted UniFrac distances at OTU level.


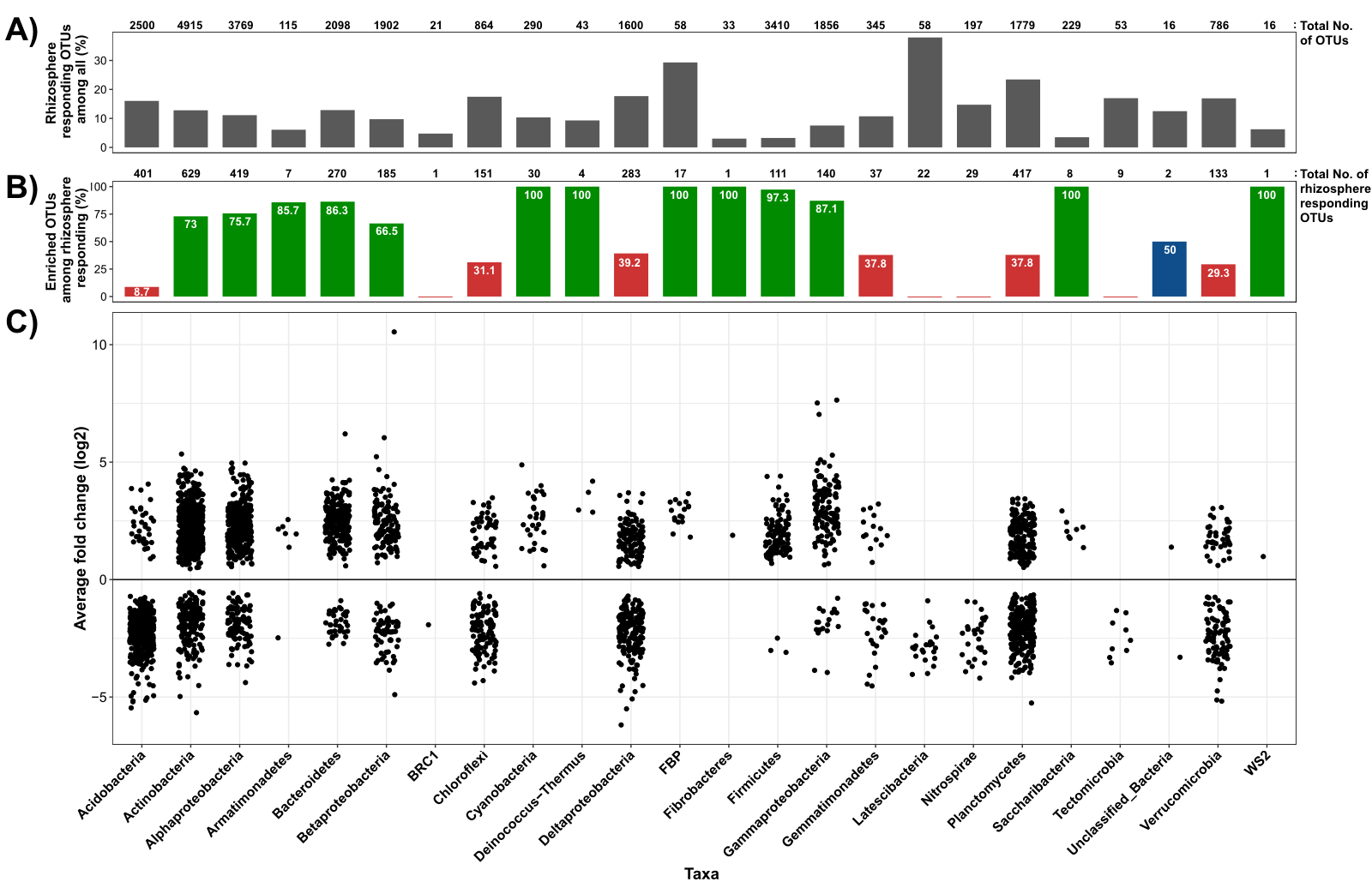


**Figure S3** Analysis of 3309 OTUs responding to the rhizosphere environment by changes in abundance. A) Percentage of rhizosphere responding OTUs among all the OTUs of a given phylum or proteobacterial class that were detected in the present study (numbers of total OTUs per phylum/class provided on top). B) Proportion of enriched OTUs (showing a significant increase in relative abundance in the rhizosphere of at least one plant species, when compared to bulk soil; as assessed by the Wald significance test and the Benjamini–Hochberg P-value correction) among all rhizosphere responding OTUs (the latter numbers are provided on top). Green bars represent phyla/classes with > 50% OTUs enriched, red bars phyla/classes with >50% OTUs depleted and blue bars represent phyla with the same number of positive and negative responders. C) Individual fold change values (log2) for each rhizosphere responding OTU.

**
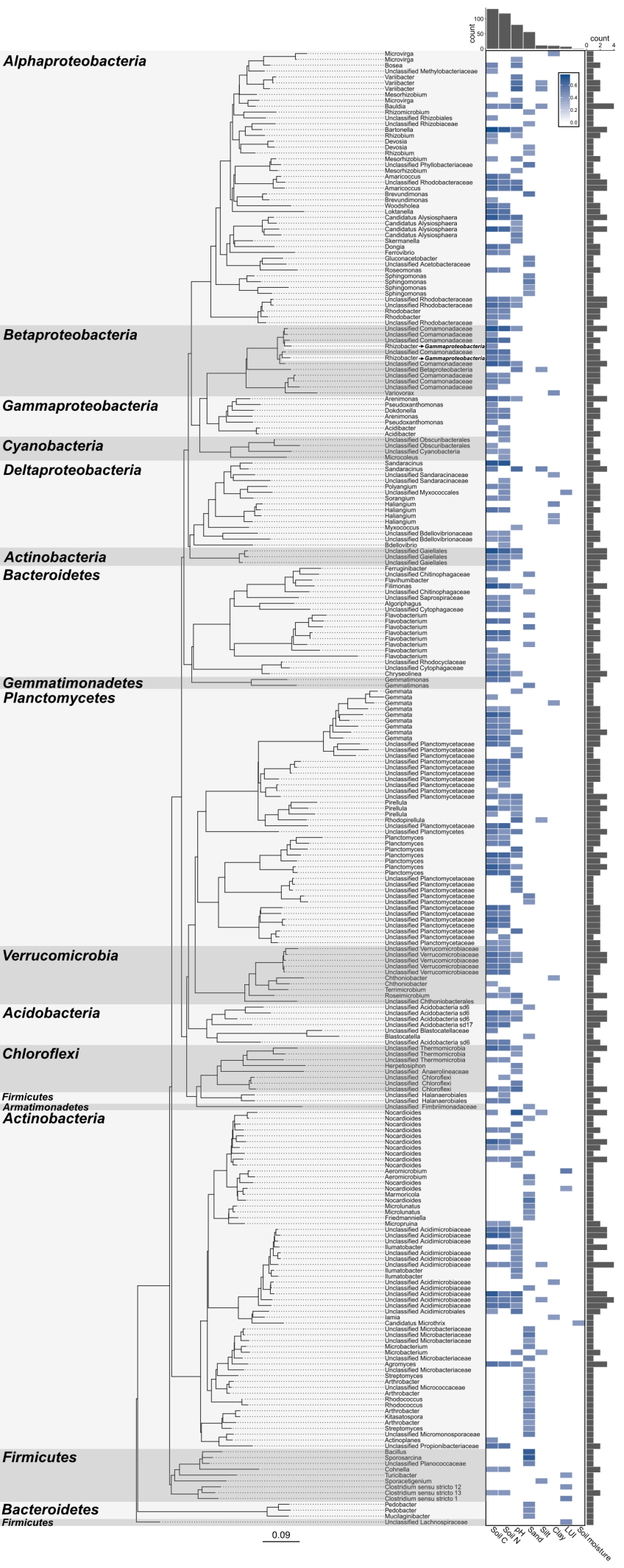
**

**Figure S4** Heatmap of significant (p < 0.01) positive correlations between rhizosphere enriched OTUs and soil parameters; with associated phylogenetic tree cropped from the SILVA SSU 128 tree. Each tip represents a single OTU labelled according to the genus name. Phyla and proteobacterial classes are indicated to the left in the shaded areas. Increasing colour depth reflects stronger correlations (only correlations R^2^>0.4 are shown). Histogram on the top shows the number of correlations registered for each soil parameter.

**
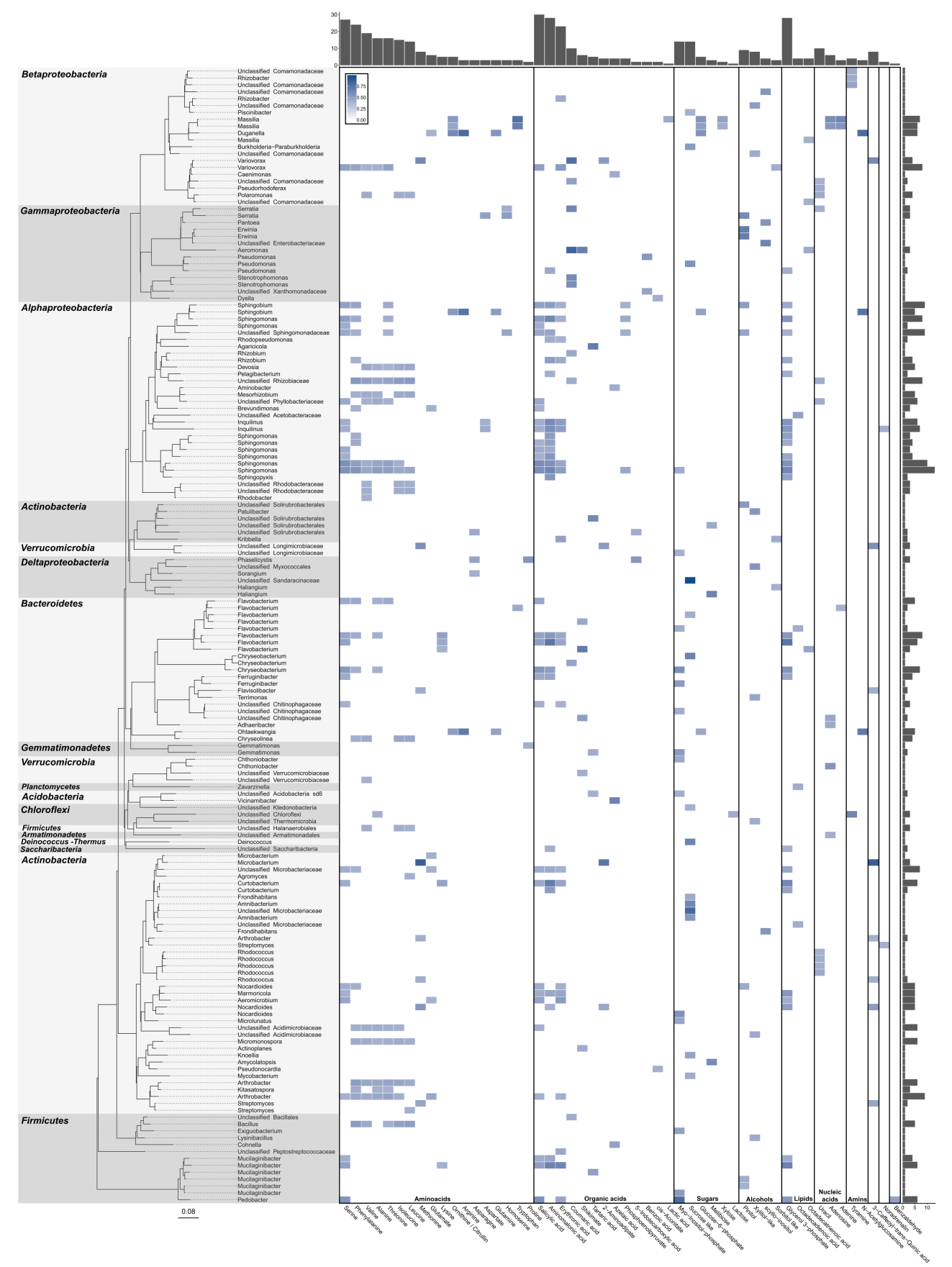
**

**Figure S5** Heatmap of significant (p < 0.01) positive correlations between rhizosphere enriched OTUs and known root exudate compounds; with associated phylogenetic tree from SILVA SSU 128, cropped to the desired OTUs. Each tip represents a single OTU labelled according to the genus name. Phyla and proteobacterial classes are indicated to the left in the shaded areas. Increasing colour depth reflects stronger correlations (only correlations R^2^>0.4 are shown). Histogram on the top shows the number of correlations registered for each root exudate.

**
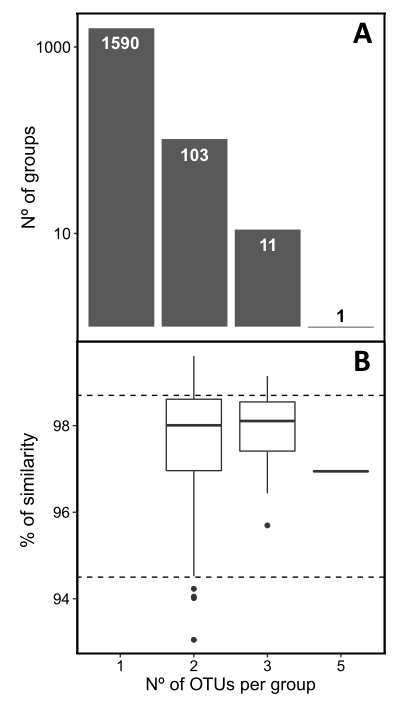
**

**Figure S6** Phylogenetic coherence of rhizosphere enriched OTUs. A) Groups of monophyletic OTUs and corresponding number of OTUs of which they were comprised. B) Percentage of sequence similarity between the members of the groups (comprised of 2 or more OTUs). For groups comprising more than 3 OTUs, mean similarity values were taken. Dotted lines reflect the current 16S rRNA sequence similarity thresholds for delineating species (98.7%) and genus (94.5%)[12].


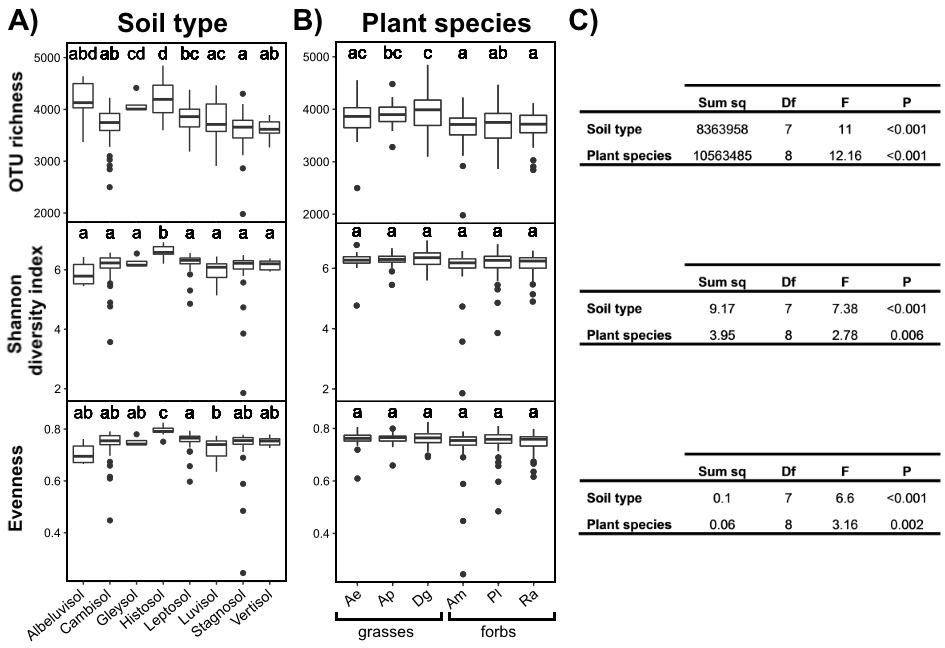


**Figure S7** α-diversity for rhizosphere bacterial communities at OTU level between A) soil types and B) plant species. Letters on top of each boxplot denote statistical significance (p < 0.05; multcomp test). Ae – *Arrenatherum elatius*; Ap – *Alopecurus pratensis*; Dg – *Dactylis glomerata*; Am – *Achillea millefolium*; Pl – *Plantago lanceolata*; Ra – *Ranunculus acris*. C) ANOVA results for the fit of a linear model for the influence of soil type and plant species on the different α-diversity metrics of rhizosphere bacterial communities.


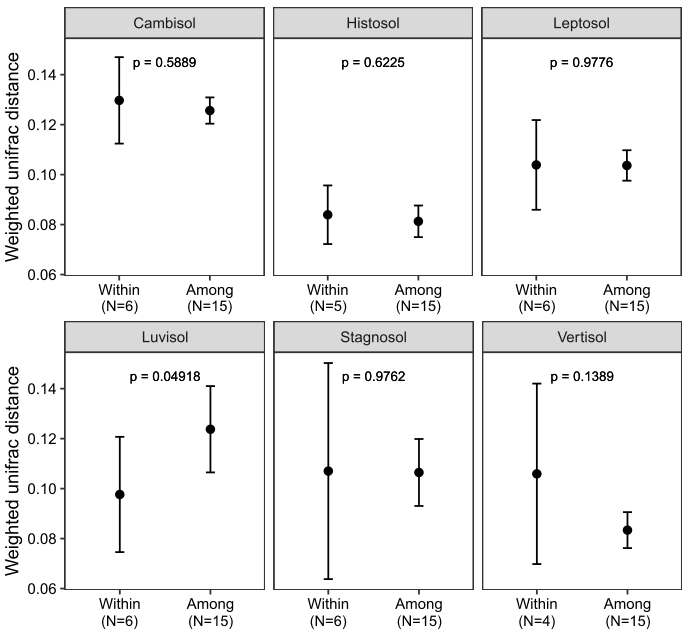


**Figure S8** Plant species have little effect on the similarity of rhizosphere bacterial communities in the same soil type. Means (black circles) and 95% confidence intervals (whiskers) of all pairwise comparisons of bacterial communities associated with a single of the six plant species (“Within”) as compared to all combinations of 2 out of 6 plant species (n=15) (“Among”). p-values were determined through t-tests. For histosols and vertisols, 1 and 2 plant species were present in only one sample and therefore no distances could be calculated for these respective plant species (hence, n=5 and n=4).


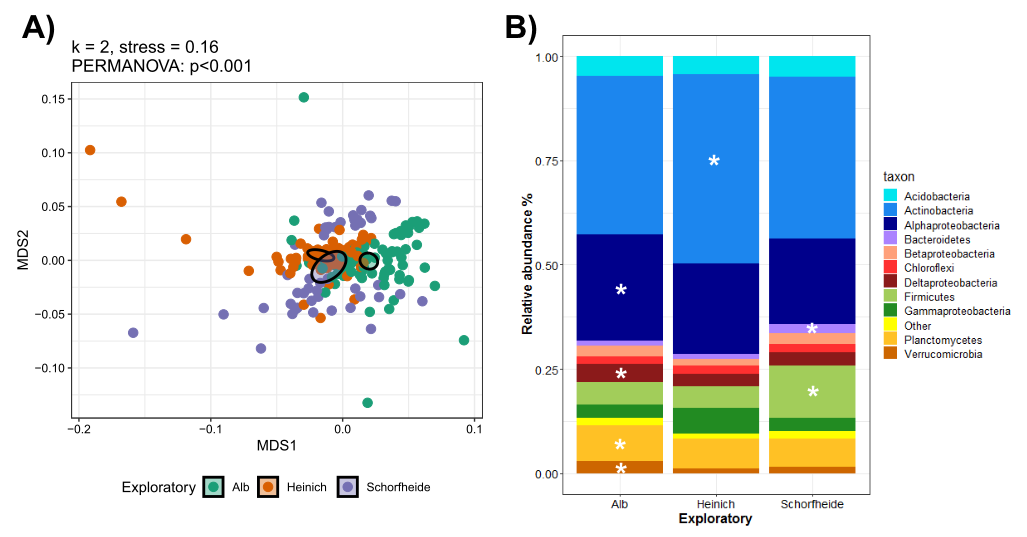


**Figure S9** Comparison of rhizosphere community structure between the three Biodiversity Exploratories regions. **A)** NMDS plot of bacterial composition based on weighted UniFrac distances at OTU level, coloured by region. Each dot represents a rhizosphere sample. Ellipses denote 95% confidence intervals. The sample for *Achillea millefolium* on plot 15 from Hainich was removed for better visualization as it represented an extreme outlier. **B)** Average relative abundances of bacterial phyla and proteobacterial classes across the regions. Taxa significantly enriched in one region when compared with the two others (multcomp test; p < 0.01) are marked with asterisks.


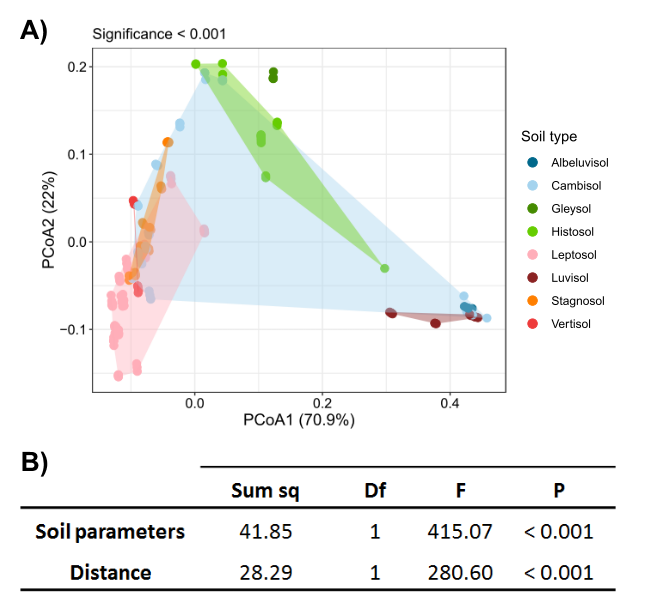


**Figure S10** Effects of spatial distance on soil variables. **A)** Principal component analysis of soil variables (Supplementary Table 3), based on Bray-Curtis distance, coloured by soil type (connected with polygons). **B)** Separation of the individual effects of the differences in soil variables and the geographic distance on the similarity between rhizosphere bacterial communities, calculated by a linear model.


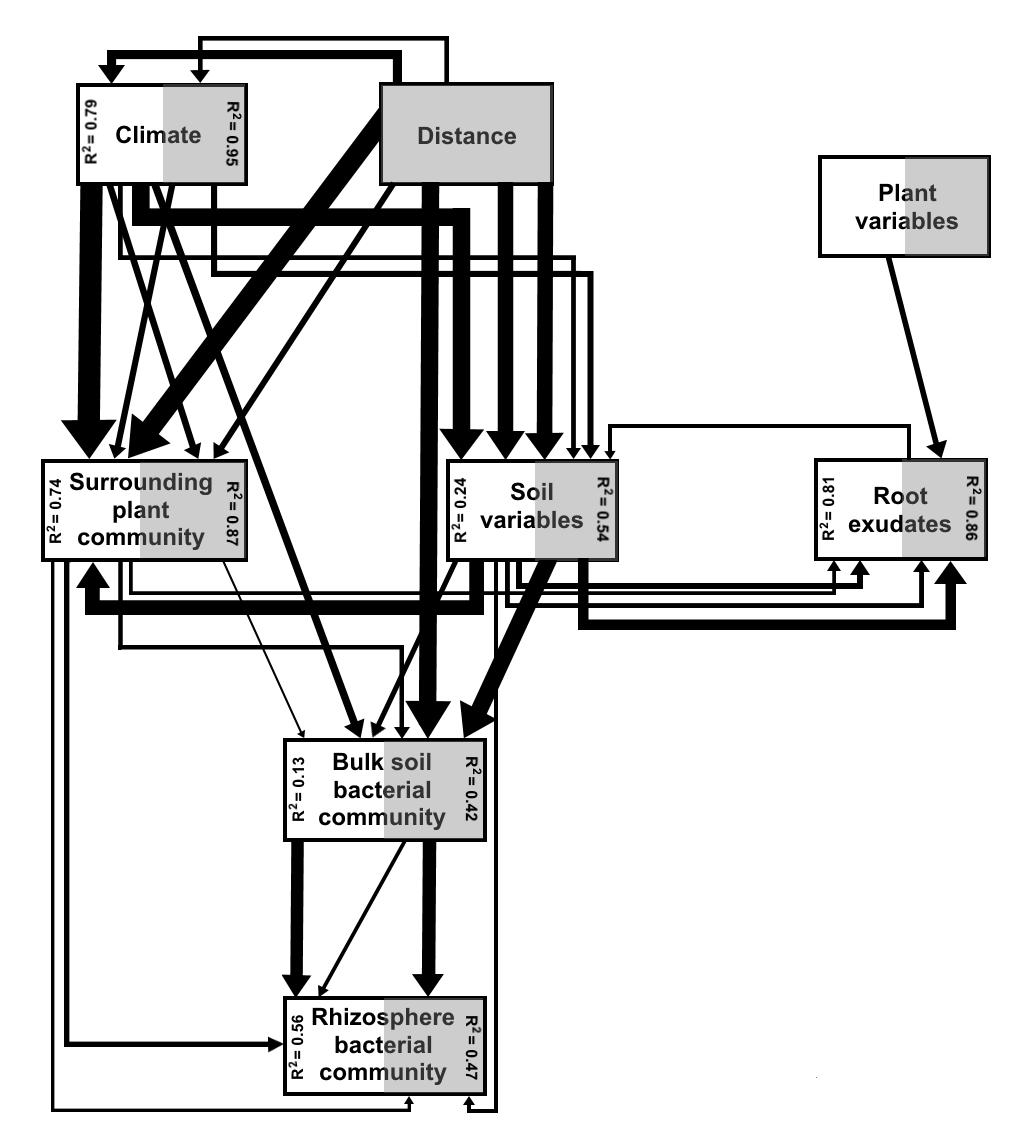


**Figure S11** Significant relationships between differences in soil variables, host plant variables, surrounding plant community, climate, spatial distance, root exudate composition, and their influence on differences in bulk soil and rhizosphere bacterial communities. White areas for each parameter represent the first axis while grey shaded areas represent the second axis of NMDS. First axis of distance is not shown as this was removed from analysis due to high correlation with the first axis of soil variables and climate. The thickness of the arrows indicates the strength of the causal relationship. R^2^ values denote the amount of variance explained by the model for the response variables. Our overall SEM model fit was satisfactory (χ2 = 40.6, P = 0.94; RMSEA = 0.000, BootstrapP = 0.94).

**Supplementary tables**

**Table S1** Number of samples analysed in each Biodiversity Exploratories region, split by soil type, plant species and plant growth conditions (natural vs phytometer; only for *Dactylis glomerata* and *Plantago lanceolata*).

|  |  | **Bulk soil** | ***Achillea***  ***millefolium*** | ***Alopecurus***  ***pratensis*** | ***Arrenatherum***  ***elatius*** | ***Ranunculus***  ***acris*** | ***Dactylis***  ***glomerata*** | | ***Plantago***  ***lanceolata*** | |
| --- | --- | --- | --- | --- | --- | --- | --- | --- | --- | --- |
| **Region** | **Soil type** |  |  |  |  |  | Phytometer | Natural | Phytometer | Natural |
| Alb | Cambisol | 4 | 1 | 2 | 4 | 3 | 4 | 3 | 4 | 4 |
|  | Leptosol | 13 | 11 | 9 | 12 | 12 | 13 | 12 | 13 | 13 |
| Hainich | Cambisol | 7 | 7 | 5 | 2 | 7 | 7 | 6 | 5 | 3 |
|  | Stagnosol | 8 | 8 | 3 | 4 | 4 | 8 | 7 | 6 | 7 |
|  | Vertisol | 2 | 1 | 2 | 2 | 2 | 2 | 2 | 1 | 2 |
| Schorfheide | Albeluvisol | 1 | 1 | 0 | 1 | 1 | 1 | 1 | 1 | 1 |
|  | Cambisol | 1 | 1 | 1 | 1 | 1 | 1 | 1 | 1 | 1 |
|  | Gleysol | 2 | 1 | 0 | 1 | 1 | 1 | 2 | 1 | 0 |
|  | Histosol | 5 | 1 | 3 | 2 | 3 | 6 | 7 | 2 | 2 |
|  | Luvisol | 3 | 3 | 2 | 2 | 2 | 3 | 4 | 3 | 4 |

**Table S2** List of primary root exudates. All annotated and identified metabolites were given with their chemical classification (class), quantifier ion (m/z) and retention time (RT in min). Retention indices (R_i_) are given for identified metabolites. Shaded compounds represent the ones used for variance partitioning.

| **Compound name** | **Class** | **Quantifier ion** | **RT (min)** | **R_i_** | **Compound name** | **Class** | **Quantifier ion** | **RT (min)** | **R_i_** | **Compound name** | **Class** | **Quantifier ion** | **RT (min)** | **R_i_** |
| --- | --- | --- | --- | --- | --- | --- | --- | --- | --- | --- | --- | --- | --- | --- |
| 2-Aminoadipate | organic acid | 260 | 23.67 | 1742.3 | unknown compound | unknown | 132 | 21.67 | - | unknown compound | unknown | 245 | 16.45 | - |
| 2-Isopropylmalate | organic acid | 275 | 20.56 | 1599.2 | unknown compound | unknown | 142 | 16.22 | - | unknown compound | unknown | 255 | 27.57 | - |
| 2-Oxoglutarate | organic acid | 129 | 16.29 | 1597.1 | unknown compound | unknown | 149 | 20.55 | - | unknown compound | unknown | 258 | 8.23 | - |
| 3-Caffeoyl-trans-quinic acid | phenylpropanoid | 345 | 47.03 | 3179 | unknown compound | unknown | 149 | 29.08 | - | unknown compound | unknown | 259 | 27.62 | - |
| 4-Aminobutanoate [GABA] | organic acid | 174 | 15.41 | 1544.7 | unknown compound | unknown | 153 | 35.95 | - | unknown compound | unknown | 259 | 31.77 | - |
| 5-Indolecarboxylic acid | organic acid | 305 | 22.84 | 2033.7 | unknown compound | unknown | 155 | 22.77 | - | unknown compound | unknown | 259 | 32 | - |
| Adenine | nucleic base | 264 | 21.31 | 1879.6 | unknown compound | unknown | 156 | 20.43 | - | unknown compound | unknown | 260 | 27.93 | - |
| Adenosine | nucleotide | 236 | 22.92 | 2680.6 | unknown compound | unknown | 158 | 8.88 | - | unknown compound | unknown | 261 | 29.85 | - |
| Adipic acid | organic acid | 111 | 15.02 | 1515.5 | unknown compound | unknown | 158 | 10.82 | - | unknown compound | unknown | 263 | 15.53 | - |
| Alanine | amino acid | 116 | 8.01 | 1113.1 | unknown compound | unknown | 158 | 15.73 | - | unknown compound | unknown | 273 | 21.48 | - |
| Aminomalonic acid | organic acid | 218 | 14.38 | 1483.7 | unknown compound | unknown | 159 | 10.83 | - | unknown compound | unknown | 273 | 21.62 | - |
| Arginine | amino acid | 157 | 15.3 | 1846.1 | unknown compound | unknown | 160 | 20.67 | - | unknown compound | unknown | 277 | 18.03 | - |
| Asparagine | amino acid | 231 | 18.07 | 1697 | unknown compound | unknown | 160 | 21.98 | - | unknown compound | unknown | 278 | 13.15 | - |
| Aspartate | amino acid | 232 | 15.32 | 1540.6 | unknown compound | unknown | 160 | 31.82 | - | unknown compound | unknown | 280 | 11.15 | - |
| Azelaic acid | organic acid | 317 | 19.64 | 1806.9 | unknown compound | unknown | 167 | 24.78 | - | unknown compound | unknown | 281 | 9.13 | - |
| Benzaldehyde | aldehyde | 257 | 17.29 | 1664.4 | unknown compound | unknown | 167 | 26.82 | - | unknown compound | unknown | 281 | 10.17 | - |
| Benzoic acid | organic acid | 267 | 16.99 | 1250.5 | unknown compound | unknown | 169 | 9.68 | - | unknown compound | unknown | 283 | 29.57 | - |
| beta-Alanine | amino acid | 248 | 13.63 | 1436.7 | unknown compound | unknown | 169 | 9.87 | - | unknown compound | unknown | 284 | 10.53 | - |
| Butylamine | amin | 202 | 8.33 | 1116.3 | unknown compound | unknown | 172 | 13.75 | - | unknown compound | unknown | 285 | 20.22 | - |
| cis-Aconitate | organic acid | 229 | 18.98 | 1770.5 | unknown compound | unknown | 173 | 30.15 | - | unknown compound | unknown | 285 | 21.83 | - |
| Coumaric acid | organic acid | 308 | 19.6 | 1807.4 | unknown compound | unknown | 173 | 7.38 | - | unknown compound | unknown | 288 | 9.83 | - |
| Erythronic acid | organic acid | 292 | 15.82 | 1571.3 | unknown compound | unknown | 173 | 11.08 | - | unknown compound | unknown | 292 | 19.43 | - |
| Fructose | sugar | 217 | 21.23 | 1913.5/1924.8 | unknown compound | unknown | 174 | 19.05 | - | unknown compound | unknown | 292 | 20.05 | - |
| Gluconate | organic acid | 333 | 23.02 | 2037.5 | unknown compound | unknown | 174 | 20.2 | - | unknown compound | unknown | 293 | 22.62 | - |
| Glucose-6-phosphate | sugar | 387 | 27.06 | 2392.9 | unknown compound | unknown | 174 | 22.12 | - | unknown compound | unknown | 295 | 19.52 | - |
| Glutamate | amino acid | 246 | 16.97 | 1643.3 | unknown compound | unknown | 174 | 7.22 | - | unknown compound | unknown | 295 | 20.98 | - |
| Glutamine | amino acid | 155 | 19.36 | 1484 | unknown compound | unknown | 174 | 15.4 | - | unknown compound | unknown | 297 | 22.87 | - |
| Glycerol 3-phosphate | lipid | 357 | 19.67 | 1799.3 | unknown compound | unknown | 174 | 15.93 | - | unknown compound | unknown | 297 | 33.7 | - |
| Homoserine | amino acid | 218 | 14.02 | 1464.4 | unknown compound | unknown | 179 | 21.2 | - | unknown compound | unknown | 299 | 21.63 | - |
| Isoleucine | amino acid | 158 | 11.28 | 1302.3 | unknown compound | unknown | 179 | 10.35 | - | unknown compound | unknown | 299 | 21.82 | - |
| Lactic acid | organic acid | 191 | 7.32 | 1076.6 | unknown compound | unknown | 179 | 14.45 | - | unknown compound | unknown | 306 | 31.08 | - |
| Lactose | sugar | 361 | 31.54 | 2738.3/2754/2768.6 | unknown compound | unknown | 185 | 25.2 | - | unknown compound | unknown | 306 | 13.87 | - |
| Leucine | amino acid | 158 | 10.98 | 1279.8 | unknown compound | unknown | 186 | 10.7 | - | unknown compound | unknown | 306 | 15.22 | - |
| Lysine | amino acid | 156 | 21.55 | 1942.9 | unknown compound | unknown | 188 | 7.83 | - | unknown compound | unknown | 311 | 22.7 | - |
| Melibiose | sugar | 361 | 32.28 | 2905.3/2917.9/2931.6 | unknown compound | unknown | 191 | 30.42 | - | unknown compound | unknown | 318 | 22.23 | - |
| Methionine | amino acid | 176 | 15.21 | 1533.4 | unknown compound | unknown | 191 | 36.25 | - | unknown compound | unknown | 319 | 21.13 | - |
| myo-Inositol | alcohol | 305 | 24.23 | 2133.3 | unknown compound | unknown | 191 | 7.25 | - | unknown compound | unknown | 319 | 21.43 | - |
| Myo-Inositol-1-phosphate | sugar | 318 | 16.89 | 2486.6 | unknown compound | unknown | 191 | 11.95 | - | unknown compound | unknown | 319 | 21.93 | - |
| N-Acetylglucosamine | amin | 156 | 19.62 | 1796.5 | unknown compound | unknown | 191 | 15.13 | - | unknown compound | unknown | 319 | 24.42 | - |
| Noradrenalin | alcaloid | 174 | 19.38 | 1759.7 | unknown compound | unknown | 192 | 10.72 | - | unknown compound | unknown | 319 | 24.43 | - |
| Octadecadienoic acid | lipid | 337 | 25.3 | 2218.9 | unknown compound | unknown | 197 | 27.28 | - | unknown compound | unknown | 319 | 24.78 | - |
| Octadecatrienoic acid | lipid | 335 | 25.21 | 2226.4 | unknown compound | unknown | 201 | 33.23 | - | unknown compound | unknown | 319 | 31 | - |
| Octadecenoic acid | lipid | 339 | 25.45 | 2223.9 | unknown compound | unknown | 201 | 8.87 | - | unknown compound | unknown | 319 | 32.8 | - |
| Ornithine / Citrullin | amino acid | 142 | 20.05 | 1843.3 | unknown compound | unknown | 204 | 21.2 | - | unknown compound | unknown | 324 | 24.27 | - |
| Phenylalanine | amino acid | 192 | 17.12 | 1650.6 | unknown compound | unknown | 204 | 22.33 | - | unknown compound | unknown | 327 | 24.27 | - |
| Phosphoenolpyruvate | sugar | 247 | 21.11 | 1624.7 | unknown compound | unknown | 204 | 25.28 | - | unknown compound | unknown | 327 | 34.42 | - |
| Pinitol | alcohol | 260 | 20.45 | 1869.4 | unknown compound | unknown | 204 | 26.72 | - | unknown compound | unknown | 331 | 24.02 | - |
| Proline | amino acid | 142 | 11.32 | 1304.4 | unknown compound | unknown | 204 | 26.92 | - | unknown compound | unknown | 331 | 24.05 | - |
| Rhamnose | sugar | 117 | 18.74 | 1756.3 | unknown compound | unknown | 204 | 27.58 | - | unknown compound | unknown | 333 | 22.08 | - |
| Ribose | sugar | 217 | 18.14 | 1709.9 | unknown compound | unknown | 204 | 28.05 | - | unknown compound | unknown | 335 | 23.08 | - |
| Salicylic acid | organic acid | 267 | 15.03 | 1518.7 | unknown compound | unknown | 204 | 28.85 | - | unknown compound | unknown | 342 | 16.6 | - |
| scyllo-inositol | alcohol | 204 | 23.33 | 2060 | unknown compound | unknown | 204 | 29.15 | - | unknown compound | unknown | 344 | 20.98 | - |
| Serine | amino acid | 204 | 12.52 | 1373.2 | unknown compound | unknown | 204 | 29.27 | - | unknown compound | unknown | 355 | 8.58 | - |
| Shikimate | organic acid | 204 | 19.99 | 1834.8 | unknown compound | unknown | 204 | 29.67 | - | unknown compound | unknown | 356 | 31.07 | - |
| Sorbitol | alcohol | 217 | 11.48 | 1315.4 | unknown compound | unknown | 204 | 29.8 | - | unknown compound | unknown | 357 | 25.43 | - |
| Succinate | organic acid | 147 | 11.56 | 1316.3 | unknown compound | unknown | 204 | 29.93 | - | unknown compound | unknown | 361 | 22.17 | - |
| Sucrose | sugar | 361 | 30.82 | 2716.8 | unknown compound | unknown | 204 | 29.97 | - | unknown compound | unknown | 361 | 31.82 | - |
| Tartaric acid | organic acid | 292 | 27.43 | 1671.9 | unknown compound | unknown | 204 | 30.57 | - | unknown compound | unknown | 361 | 31.98 | - |
| Threitol | alcohol | 217 | 15.33 | 1525.4 | unknown compound | unknown | 204 | 30.88 | - | unknown compound | unknown | 361 | 32.1 | - |
| Threonine | amino acid | 218 | 13.01 | 1401.7 | unknown compound | unknown | 204 | 31.7 | - | unknown compound | unknown | 361 | 32.47 | - |
| Tryptophan | amino acid | 202 | 25.66 | 2244.5 | unknown compound | unknown | 204 | 31.9 | - | unknown compound | unknown | 361 | 33.38 | - |
| Tyramine | amin | 174 | 21.36 | 1926.5 | unknown compound | unknown | 204 | 32.32 | - | unknown compound | unknown | 361 | 34.22 | - |
| Tyrosine | amino acid | 218 | 21.89 | 1961.1 | unknown compound | unknown | 204 | 32.63 | - | unknown compound | unknown | 362 | 32.97 | - |
| Uracil | nucleic base | 241 | 12.14 | 1344.9 | unknown compound | unknown | 204 | 32.85 | - | unknown compound | unknown | 362 | 34.52 | - |
| Valine | amino acid | 144 | 9.89 | 1222.4 | unknown compound | unknown | 204 | 33.1 | - | unknown compound | unknown | 525 | 33.2 | - |
| Xylitol | alcohol | 307 | 18.59 | 1735.6 | unknown compound | unknown | 204 | 33.52 | - | unknown compound | unknown | 56 | 27.92 | - |
| Xylose | sugar | 217 | 17.67 | 1685.4/1694.4 | unknown compound | unknown | 204 | 34.47 | - | unknown compound | unknown | 57 | 18.78 | - |
| unknown sugar | unknown sugar | 204 | 21.9 | - | unknown compound | unknown | 204 | 35.78 | - | unknown compound | unknown | 57 | 19.55 | - |
| unknown sugar | unknown sugar | 204 | 22.02 | - | unknown compound | unknown | 204 | 37.07 | - | unknown compound | unknown | 57 | 21.9 | - |
| unknown sugar | unknown sugar | 319 | 29.68 | - | unknown compound | unknown | 204 | 38.82 | - | unknown compound | unknown | 57 | 27.58 | - |
| unknown sugar | unknown sugar | 319 | 31.88 | - | unknown compound | unknown | 205 | 22.45 | - | unknown compound | unknown | 57 | 35.73 | - |
| unkown fatty acid | unknown lipid | 339 | 25.3 | - | unknown compound | unknown | 205 | 32.1 | - | unknown compound | unknown | 58 | 7.83 | - |
| unknown compound | unknown | 103 | 17.58 | - | unknown compound | unknown | 205 | 10.88 | - | unknown compound | unknown | 69 | 19 | - |
| unknown compound | unknown | 103 | 18.88 | - | unknown compound | unknown | 207 | 7.27 | - | unknown compound | unknown | 69 | 19.17 | - |
| unknown compound | unknown | 103 | 19.43 | - | unknown compound | unknown | 207 | 7.57 | - | unknown compound | unknown | 69 | 19.52 | - |
| unknown compound | unknown | 103 | 20.3 | - | unknown compound | unknown | 216 | 29.8 | - | unknown compound | unknown | 71 | 20.95 | - |
| unknown compound | unknown | 103 | 20.72 | - | unknown compound | unknown | 217 | 18.72 | - | unknown compound | unknown | 71 | 21.3 | - |
| unknown compound | unknown | 103 | 21.05 | - | unknown compound | unknown | 217 | 19.25 | - | unknown compound | unknown | 71 | 21.93 | - |
| unknown compound | unknown | 103 | 21.07 | - | unknown compound | unknown | 217 | 19.43 | - | unknown compound | unknown | 71 | 15.57 | - |
| unknown compound | unknown | 103 | 13.38 | - | unknown compound | unknown | 217 | 19.67 | - | unknown compound | unknown | 74 | 10.53 | - |
| unknown compound | unknown | 103 | 16.23 | - | unknown compound | unknown | 217 | 21.2 | - | unknown compound | unknown | 75 | 20.3 | - |
| unknown compound | unknown | 103 | 16.37 | - | unknown compound | unknown | 217 | 24.05 | - | unknown compound | unknown | 75 | 7.25 | - |
| unknown compound | unknown | 112 | 7.52 | - | unknown compound | unknown | 217 | 29.18 | - | unknown compound | unknown | 75 | 11.5 | - |
| unknown compound | unknown | 116 | 7.9 | - | unknown compound | unknown | 217 | 31.28 | - | unknown compound | unknown | 75 | 12.03 | - |
| unknown compound | unknown | 117 | 17.23 | - | unknown compound | unknown | 217 | 32.27 | - | unknown compound | unknown | 75 | 12.28 | - |
| unknown compound | unknown | 117 | 17.28 | - | unknown compound | unknown | 217 | 33.23 | - | unknown compound | unknown | 75 | 13.73 | - |
| unknown compound | unknown | 117 | 18.68 | - | unknown compound | unknown | 217 | 33.77 | - | unknown compound | unknown | 77 | 7.77 | - |
| unknown compound | unknown | 117 | 25.53 | - | unknown compound | unknown | 217 | 16.07 | - | unknown compound | unknown | 82 | 28.33 | - |
| unknown compound | unknown | 117 | 30.05 | - | unknown compound | unknown | 219 | 29.6 | - | unknown compound | unknown | 83 | 27.38 | - |
| unknown compound | unknown | 117 | 7.38 | - | unknown compound | unknown | 219 | 10.27 | - | unknown compound | unknown | 83 | 32.9 | - |
| unknown compound | unknown | 117 | 7.65 | - | unknown compound | unknown | 223 | 32.28 | - | unknown compound | unknown | 87 | 7.25 | - |
| unknown compound | unknown | 117 | 8.82 | - | unknown compound | unknown | 223 | 16.38 | - | unknown compound | unknown | 89 | 7.32 | - |
| unknown compound | unknown | 117 | 10.08 | - | unknown compound | unknown | 227 | 15.75 | - | unknown compound | unknown | 89 | 7.33 | - |
| unknown compound | unknown | 119 | 33.65 | - | unknown compound | unknown | 229 | 13.97 | - | unknown compound | unknown | 89 | 9.58 | - |
| unknown compound | unknown | 120 | 15.67 | - | unknown compound | unknown | 237 | 14.18 | - | unknown compound | unknown | 89 | 11.77 | - |
| unknown compound | unknown | 125 | 8.47 | - | unknown compound | unknown | 239 | 27.38 | - | unknown compound | unknown | 91 | 27.67 | - |
| unknown compound | unknown | 126 | 11.17 | - | unknown compound | unknown | 241 | 9.22 | - | unknown compound | unknown | 91 | 34.25 | - |
| unknown compound | unknown | 127 | 29.67 | - | unknown compound | unknown | 243 | 13.83 | - | unknown compound | unknown | 91 | 34.95 | - |
| unknown compound | unknown | 127 | 7.83 | - | unknown compound | unknown | 245 | 17.48 | - | unknown compound | unknown | 93 | 18.87 | - |
| unknown compound | unknown | 128 | 25.07 | - | unknown compound | unknown | 245 | 17.75 | - |  |  |  |  |  |

**Table S3 List of plant and soil variables and of surrounding plant community variables associated with each rhizosphere sample.**

| **Plant parameters** | **Units** |
| --- | --- |
| Plant species | *Arrenatherum elatius*; *Achillea millefolium*; *Alopecurus pratensis*; *Dactilys glomerata*; *Plantago lanceolata*; *Ranunculus acris* |
| Plant growth form | forb or grass |
| Root fresh mass | g |
| Plant fresh mass | g |
| Diameter of plant | mm (2 perpendicular measurements) |
| Height of plant, elongated | mm |
| Leaves | N° |
| Root carbon content | % |
| Root nitrogen content | % |
| **Soil parameters** | **Units** |
| Soil type | Albeluvisol, Cambisol, Gleysol, Luvisol, Leptosol, Histosol, Stagnosol, Vertisol |
| Exploratory region | Swäbische-Alb; Hainich-Dün; Schorfheide-Chorin |
| Land Use Intensity Index (LUI) | * |
| pH | 0-14 |
| Soil carbon content | g.kg^-1^ |
| Soil nitrogen content | g.kg^-1^ |
| Soil moisture at 10cm depth | % (volumetric water content; average of monthly mean values from May 2014 to July 2015) |
| Soil clay content | g.kg^-1^ |
| Soil silt content | g.kg^-1^ |
| Soil sand content | g.kg^-1^ |
| **Climate** | **Units** |
| Air temperature 10 cm aboveground | °C (average of monthly mean values from May 2014 to July 2015) |
| Air temperature 2 m aboveground | °C (average of monthly mean values from May 2014 to July 2015) |
| Relative humidity 2 m above ground | % (average of monthly mean values from May 2014 to July 2015) |
| Precipitation | mm (average of monthly mean values from May 2014 to July 2015) |
| **Surrounding plant community** | **Units** |
| Plant species in the 15 cm radius around each phytometer | *Achillea millefolium*, *Agrimonia eupatoria*, *Alchemilla* spec., *Alopecurus pratensis*, *Anthoxanthum odoratum*, *Anthriscus sylvestris*, *Anthyllis vulneraria*, *Arctium minus*, *Arrhenatherum elatius*, *Asperula cynanchica*, *Brachypodium pinnatum*, *Briza media*, *Bromus erectus*, *Bromus hordeaceus*, *Campanula patula*, *Campanula rotundifolia*, *Cardamine hirsute*, *Carex caryophyllea*, *Carex flacca*, *Carex hirta*, *Carex* spec., *Carum carvi*, *Centaurea jacea*, *Cerastium holosteoides*, *Cirsium acaule*, *Cirsium arvense, Convolvulus arvensis, Crepis biennis, Cruciata laevipes, Cynosurus cristatus, Dactylis glomerata, Daucus carota, Deschampsia cespitosa, Elymus repens, Euphorbia cyparissias, Euphorbia verrucosa, Euphrasia officinalis, Falcaria vulgaris, Festuca guestfalica, Festuca ovina, Festuca pratensis, Festuca rubra, Galium mollugo* agg.*, Galium pumilum, Galium verum, Gentiana verna, Geranium pratense, Glechoma hederacea, Helianthemum nummularium, Helictotrichon pratense, Helictotrichon pubescens, Heracleum sphondylium, Hieracium pilosella, Hieracium* spec.*, Holcus lanatus, Knautia arvensis, Koeleria macrantha, Koeleria pyramidata, Lathyrus pratensis, Leontodon autumnalis, Leontodon hispidus, Leucanthemum vulgare, Linum catharticum, Lolium multiflorum, Lolium perenne, Lotus corniculatus, Luzula campestris, Medicago lupulina, Myosotis arvensis, Ononis repens, Phleum pratense, Pimpinella saxifraga, Plantago lanceolata, Plantago major, Plantago media, Poa pratensis, Poa trivialis, Polygala vulgaris, Potentilla anserina, Potentilla heptaphylla, Potentilla reptans, Primula veris, Prunella grandiflora, Prunella vulgaris, Prunus spinosa, Ranunculus acris, Ranunculus bulbosus, Ranunculus repens, Rhinanthus alectorolophus, Rhinanthus minor, Rumex acetosa, Salvia pratensis, Sanguisorba minor, Scabiosa columbaria, Senecio jacobaea, Stellaria graminea, Taraxacum Sec. Ruderalia, Teucrium montanum, Thlaspi arvense, Thymus pulegioides, Tragopogon pratensis, Trifolium campestre, Trifolium pratense, Trifolium repens, Trisetum flavescens, Veronica arvensis, Veronica chamaedrys, Veronica serpyllifolia, Vicia sepium, Vicia* spec.. |
| Area covered by each plant species in15 cm radius around each phytometer | % |

*****See Blüthgen *et al.*, 2012 [2]

**Table S4** – Top 20 most abundant rhizosphere enriched OTUs (Average relative abundance of an OTU in the rhizosphere is 0.004). Shaded OTUs belong to genera with members known for their plant growth promoting capacities.

| **Average relative abundance in rhizosphere %** | **OTU** | **Phylum** | **Class** | **Order** | **Family** | **Genera** |
| --- | --- | --- | --- | --- | --- | --- |
| 1.57 | AJ316140.1.1445 | *Actinobacteria* | *Actinobacteria* | *Streptomycetales* | *Streptomycetaceae* | *Streptomyces* |
| 1.35 | JF167768.1.1343 | *Actinobacteria* | *Actinobacteria* | *Streptomycetales* | *Streptomycetaceae* | *Streptomyces* |
| 1.33 | HM057824.1.1347 | *Proteobacteria* | *Gammaproteobacteria* | *Pseudomonadales* | *Pseudomonadaceae* | *Pseudomonas* |
| 1.21 | EF018802.1.1366 | *Actinobacteria* | *Actinobacteria* | *Frankiales* | *Nakamurellaceae* | *Nakamurella* |
| 1.02 | KF098235.1.1340 | *Actinobacteria* | *Actinobacteria* | *Propionibacteriales* | *Propionibacteriaceae* | *Microlunatus* |
| 0.91 | DQ870743.1.1232 | *Actinobacteria* | *Actinobacteria* | *Micrococcales* | *Microbacteriaceae* | *Microbacterium* |
| 0.84 | AJ919996.1.1482 | *Actinobacteria* | *Actinobacteria* | *Micrococcales* | *Micrococcaceae* | *Paenarthrobacter* |
| 0.79 | AF434185.1.1467 | *Actinobacteria* | *Actinobacteria* | *Micrococcales* | *Micrococcaceae* | *Paenarthrobacter* |
| 0.78 | DQ906897.1.1409 | *Proteobacteria* | *Alphaproteobacteria* | *Rhizobiales* | *Methylobacteriaceae* | *Microvirga* |
| 0.72 | AJ278249.1.1466 | *Proteobacteria* | *Alphaproteobacteria* | *Rhizobiales* | *Phyllobacteriaceae* | *Mesorhizobium* |
| 0.72 | EF191199.1.1418 | *Actinobacteria* | *Actinobacteria* | *Micromonosporales* | *Micromonosporaceae* | *-* |
| 0.59 | AJ536673.1.1406 | *Proteobacteria* | *Alphaproteobacteria* | *Rhizobiales* | *Phyllobacteriaceae* | *Mesorhizobium* |
| 0.59 | KR560013.1.1478 | *Firmicutes* | *Bacilli* | *Bacillales* | *Bacillaceae* | *Bacillus* |
| 0.57 | AF408945.1.1377 | *Actinobacteria* | *Actinobacteria* | *Micrococcales* | *Intrasporangiaceae* | *Terrabacter* |
| 0.53 | AF423291.1.1411 | *Proteobacteria* | *Alphaproteobacteria* | *Sphingomonadales* | *Sphingomonadaceae* | *Sphingomonas* |
| 0.52 | AJ399492.1.1450 | *Actinobacteria* | *Actinobacteria* | *Micrococcales* | *Cellulomonadaceae* | *Cellulomonas* |
| 0.50 | EU240416.1.1346 | *Actinobacteria* | *Actinobacteria* | *Micromonosporales* | *Micromonosporaceae* | *Dactylosporangium* |
| 0.41 | GQ016330.1.1340 | *Actinobacteria* | *Acidimicrobiia* | *Acidimicrobiales* | *Acidimicrobiaceae* | *Ilumatobacter* |
| 0.41 | AF539697.1.1431 | *Actinobacteria* | *Actinobacteria* | *Micrococcales* | *Microbacteriaceae* | *-* |
| 0.41 | AM743175.1.1453 | *Proteobacteria* | *Alphaproteobacteria* | *Rhodospirillales* | *Rhodospirillaceae* | *Skermanella* |

**Table S5** – Top 20 known genera with highest numbers of rhizosphere enriched OTUs. Shaded genera include members known for their plant growth promoting capacities.

| **Average relative abundance in rhizosphere %** | **N° of OTUs** | **Genera** |
| --- | --- | --- |
| 3.31 | 61 | *Nocardioides* |
| 3.12 | 25 | *Streptomyces* |
| 2.43 | 38 | *Pseudomonas* |
| 1.69 | 16 | *Microbacterium* |
| 1.66 | 4 | *Paenarthrobacter* |
| 1.57 | 19 | *Bacillus* |
| 1.45 | 9 | *Nakamurella* |
| 1.44 | 8 | *Microlunatus* |
| 1.39 | 11 | *Mesorhizobium* |
| 1.34 | 7 | *Microvirga* |
| 1.02 | 26 | *Sphingomonas* |
| 0.99 | 4 | *Cellulomonas* |
| 0.79 | 2 | *Terrabacter* |
| 0.59 | 47 | *Gemmata* |
| 0.59 | 10 | *Devosia* |
| 0.57 | 7 | *Candidatus Alysiosphaera* |
| 0.56 | 7 | *Aeromicrobium* |
| 0.52 | 18 | *Mycobacterium* |
| 0.52 | 16 | *Rhizobium* |
| 0.5 | 1 | *Dactylosporangium* |
